# A conceptual framework for quality assessment and management of biodiversity data

**DOI:** 10.1371/journal.pone.0178731

**Published:** 2017-06-28

**Authors:** Allan Koch Veiga, Antonio Mauro Saraiva, Arthur David Chapman, Paul John Morris, Christian Gendreau, Dmitry Schigel, Tim James Robertson

**Affiliations:** 1 University of São Paulo, Research Center on Biodiversity and Computing, São Paulo, São Paulo, Brazil; 2 Australian Biodiversity Information Services, Ballan, Victoria, Australia; 3 Harvard University, Museum of Comparative Zoology, Cambridge, Massachusetts, United States of America; 4 Université de Montréal, Institut de Recherche en Biologie Végétale, Montréal, Québec, Canada; 5 Global Biodiversity Information Facility, Secretariat, Copenhagen, Denmark; Oklahoma State University, UNITED STATES

## Abstract

The increasing availability of digitized biodiversity data worldwide, provided by an increasing number of institutions and researchers, and the growing use of those data for a variety of purposes have raised concerns related to the "fitness for use" of such data and the impact of data quality (DQ) on the outcomes of analyses, reports, and decisions. A consistent approach to assess and manage data quality is currently critical for biodiversity data users. However, achieving this goal has been particularly challenging because of idiosyncrasies inherent in the concept of quality. DQ assessment and management cannot be performed if we have not clearly established the quality needs from a data user’s standpoint. This paper defines a formal conceptual framework to support the biodiversity informatics community allowing for the description of the meaning of "fitness for use" from a data user’s perspective in a common and standardized manner. This proposed framework defines nine concepts organized into three classes: DQ Needs, DQ Solutions and DQ Report. The framework is intended to formalize human thinking into well-defined components to make it possible to share and reuse concepts of DQ needs, solutions and reports in a common way among user communities. With this framework, we establish a common ground for the collaborative development of solutions for DQ assessment and management based on data fitness for use principles. To validate the framework, we present a proof of concept based on a case study at the Museum of Comparative Zoology of Harvard University. In future work, we will use the framework to engage the biodiversity informatics community to formalize and share DQ profiles related to DQ needs across the community.

## 1. Introduction

Data Quality (DQ) is a subject that permeates most research. As a result, research on DQ, information quality, or data fitness for use has been conducted and applied in a number of domains, covering multiple aspects and approaches [1–6].

Most DQ investigations can be classified into three branches: DQ assessment, DQ management and contextual DQ [1]. Research on DQ assessment aims at the measurement, validation and classification of the quality of data [7–11]. DQ management research aims at the improvement of the usefulness of data through error prevention and correction by quality control or quality assurance [8, 12, 13]. Contextual DQ research aims at the evaluation of the impact of DQ in organizations [2, 14–16].

Biodiversity sciences are one of those data intensive research fields that demand investigations on DQ, especially on DQ assessment and management. Biodiversity informatics aims at applying computing concepts, techniques and tools to research on biological diversity [17–24]. During the past 40 years, much effort has been applied to enable the digitization, integration and sharing of distributed biodiversity data. Biodiversity data are stored in heterogeneous formats and spread across many providers around the world, such as museums, botanical gardens, universities, and other institutions holding biological collections and related data [25–32].

To support the international collaboration of biodiversity informatics institutions and projects, Biodiversity Information Standards (TDWG, from its former name Taxonomic Databases Working Group) was established to develop, adopt and promote standards and guidelines for the recording and exchange of data about organisms [33]. Standards and protocols developed, adopted and promoted by TDWG have enabled many initiatives to create efficient platforms for the interoperability, integration and publishing of biodiversity data around the world.

The largest platform in number of published records of species occurrences in the world is the Global Biodiversity Information Facility (GBIF) [34]. GBIF has delivered an international open data infrastructure for encouraging and helping institutions to aggregate and publish data according to common standards and allowing anyone, anywhere to access data about all types of life on Earth, shared across national boundaries via the Internet [35]. By 2017, GBIF had aggregated and enabled access to more than seven hundred million occurrence records provided from more than 50 countries. These published data are being used for an expanding range of applications as more users become aware of the significant resource that is available [3].

Such data aggregation initiatives, however, have highlighted DQ problems, which, despite the long-standing focus on standards and data quality in the domain (e.g. [19, 27]), are common and present in much biodiversity data from providers around the world. Several efforts for determining and improving DQ have been proposed and put into practice (some examples include [3, 36–44]). To date, no consistent framework has been developed for describing, assessing and managing the quality of biodiversity data; thus, it is difficult for users of such data to adequately compare the quality of one dataset to another and determine the fitness of data for their particular uses. In other words, DQ needs are common, but formal language to express those in a comparable and repeatable way has been lacking.

Inadequacies of quality assessment and quality management of biodiversity data are currently highlighted by the use of the data for modeling of species distributions. The quality of a significant amount of the available data is currently not satisfactory or is undetermined for many of the desired uses [45, 46]. To enable the DQ assessment and management of biodiversity data, it is necessary to define relevant components to allow biodiversity data users, curators, holders and owners to determine and improve the fitness of data for use.

We define **DQ assessment** as the action of judging the data fitness for use in a given context. The assessment can be performed in two ways; (1) **Quantitatively**: the evaluation of the level of fitness for a specific use; (2) **Qualitatively**: the judgment whether the quality of data is or is not fit for a specific use. It is important not to confuse DQ assessment with DQ measurement. DQ assessment uses a set of quality measurements to determine the fitness for use, but data with the same quality measures may have different levels of fitness for different uses. Broadly, three interrelated components are necessary to determine **data fitness for use**:

- **Use**: What is the purpose or use for which data must have quality?
- **Data**: What kind of data are relevant and must have quality in the context of the Use?
- **Fitness**: What constitutes “fitness” for the relevant Data in the context of the Use?

**DQ management** is defined as the action of improving the quality of data and, consequently, making data fitter for use for a particular purpose. DQ management can be performed using two approaches:

- **DQ Control**: aims to improve the quality of data by preventing errors, correcting errors or proposing corrections;
- **DQ Assurance**: aims to ensure that data selected for use have satisfactory quality for a particular purpose; this approach implies filtering and excluding data which lack the required quality for the purpose.

This paper introduces a conceptual framework to define and organize the necessary concepts for enabling the assessment and management of the data fitness for use in the domain of biodiversity informatics. The design of the conceptual framework draws from the DQ literature [1, 47, 48]. The framework organizes relevant concepts to tackle DQ issues in the context of biodiversity informatics, specifically scenarios that deal with distributed data, encompassing a diverse community of users and a wide range of data usages, and where DQ requirements may not be clearly and easily defined, accepted and standardized universally. Inherent in biodiversity, particularly in taxonomy, is the complexity of differing opinions about acceptable sources of truth (e.g. [49]).

Based on the DQ research area, this framework is consistent with several DQ models available in the literature [1], such as TDQM [50]. TDMQ is a comprehensive and structured approach to organizational management for improving the DQ [12, 51, 52].

**Fig 1:**
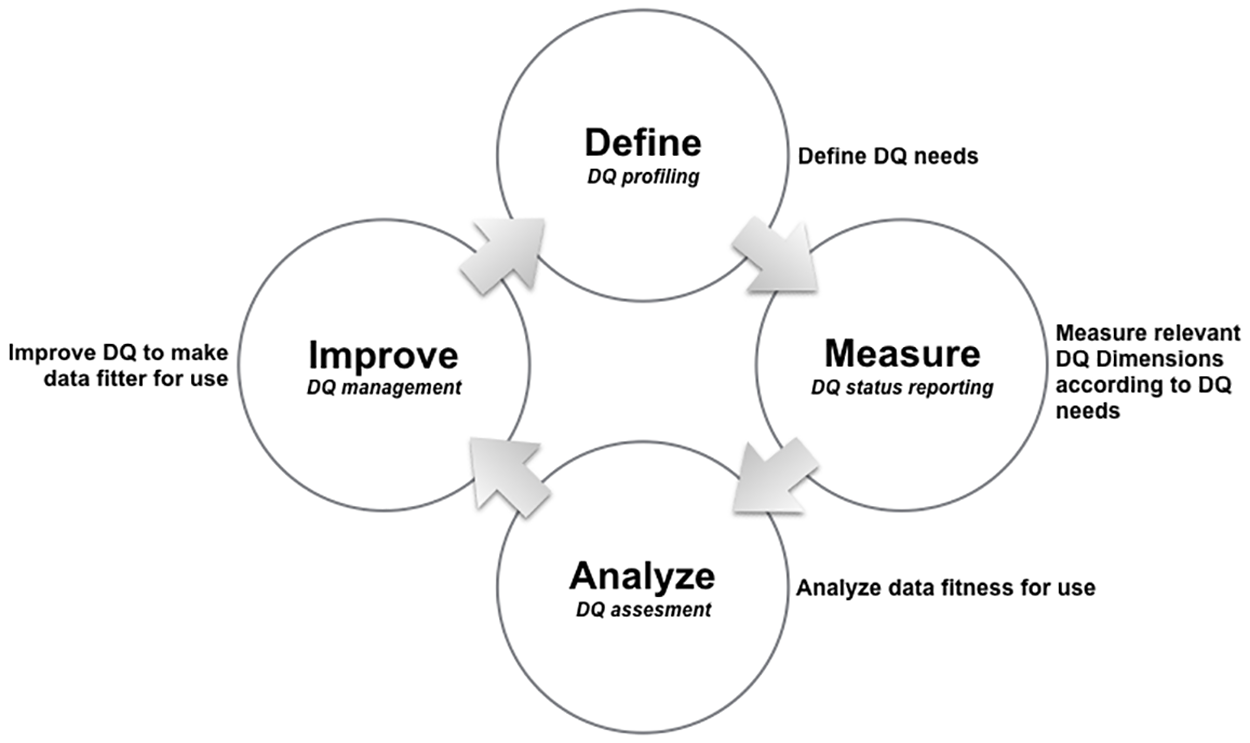
The relationship between TDQM steps and the proposed conceptual framework. The relationship between TDQM steps and the conceptual framework is given by the following related components: (1) Define—DQ profiling; (2) Measure—DQ status reporting; (3) Analyze—DQ assessment; and (4) Improve—DQ management.

TDQM is organized into four main steps: (1) define, (2) measure, (3) analyze and (4) improve, as illustrated in Fig 1. The first step defines the DQ needs in a given context. In the second step, the DQ is measured according to the defined DQ needs. The third step intends to analyze whether the quality of data is suitable for use. The fourth step implements actions to improve the measures of quality making data fitter for use.

In the conceptual framework context, the TDQM first step is related to DQ profiling (see Sections 2.1 and 3.1), the second step to DQ status reporting (see Sections 2.2, 2.3 and 3.2), the third step to the DQ assessment (the action of judging the fitness for use) and fourth step to DQ management (the action of improving DQ, making data fitter for use).

To validate the conceptual framework, we present a proof of concept that addresses the framework concepts and their application to enable the assessment and management of fitness for use of a real dataset. Details of the proof of concept are available in S1 Appendix. For quick reference we compile most of the main terms used in this work in S2 Appendix.

The framework, presented in Section 2, introduces the components for formalizing the DQ needs/solutions to generate relevant reports with quality measures, validations and amendments, allowing users to draw conclusions concerning the quality of datasets and single records for particular purposes, as presented in Section 3.

## 2. Conceptual framework

The Biodiversity Informatics community has successfully supported initiatives to deliver platforms for the free access to biodiversity data integrated from many data providers around the world in a standardized and common way. However, before using such data, the data user has to ask if such available data are fit for their particular use.

**Fig 2:**
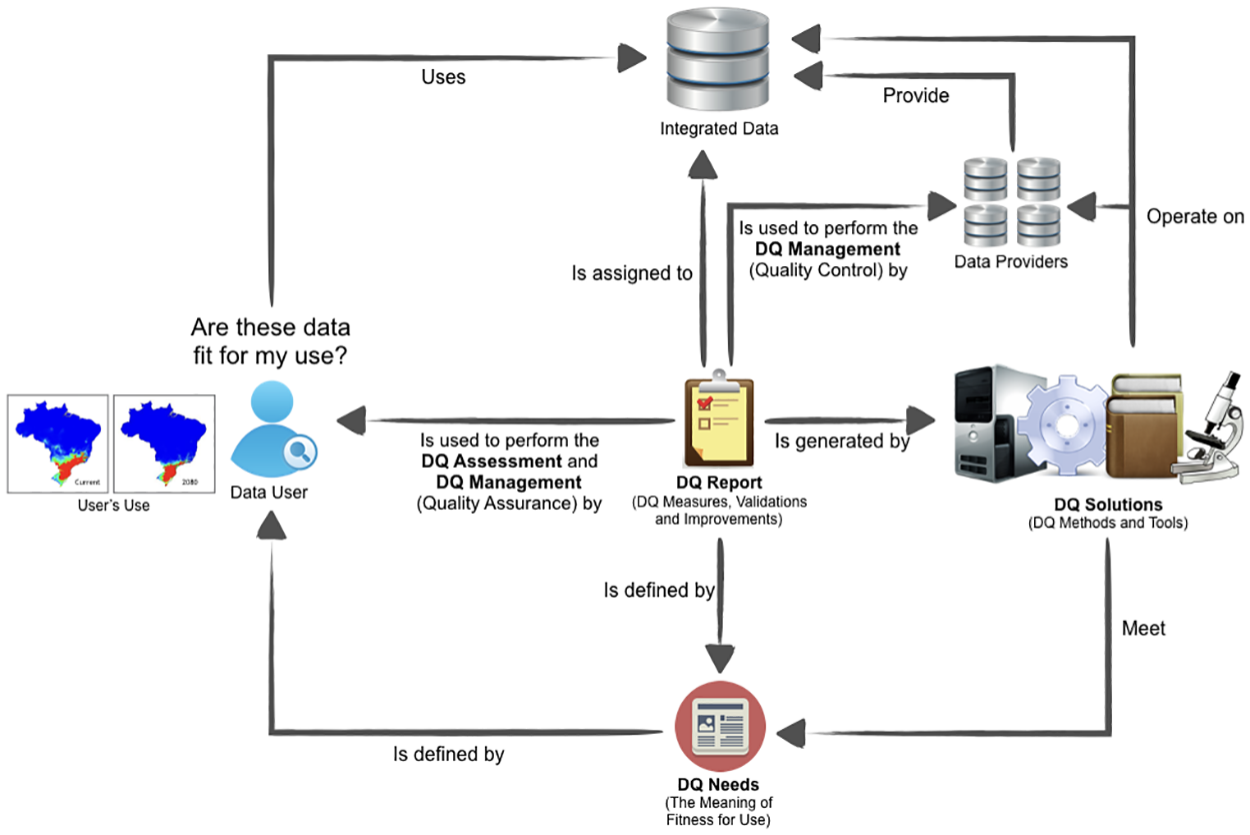
Components to enable the DQ assessment and management in the context of biodiversity informatics. Three main components are necessary to enable DQ assessment and management: (1) DQ needs according to data users, (2) DQ solutions to meet the defined DQ needs and (3) DQ reports presenting the status of quality of data according to data users perspective.

To answer that question, it is necessary to define the **DQ needs** according to data users as illustrated in Fig 2. Due to the idiosyncratic nature of the concept of “quality,” it is essential to understand what "data fitness for use" means according to the data user’s perspective to enable the DQ assessment and management.

Given some user’s DQ needs, DQ solutions must be delivered to meet those defined DQ needs. **DQ solutions** are anything able to perform DQ measures, validations or amendments in datasets or single records. The results obtained by the DQ solutions must be reported.

A **DQ report** is a set of DQ assertions assigned to a dataset or a single record, generated by DQ solutions according to users’ DQ needs. The DQ report describes the current status of quality of a dataset or a single record according to the perspectives of data users. Such report contains DQ measures, validations and amendments that enable data users to perform the DQ assessment and, perhaps, the selection of a subset of the data from the original dataset that is fit for use, i.e. to perform the DQ management by the DQ assurance approach. A DQ report can also be used by data providers to improve their own data by accepting proposed amendments as changes to their data, and by evaluating proposed amendments and identifying user training needs or systemic needs of their information management systems, or by just highlighting the quality of the data, that is, to perform DQ management by the DQ control approach. See more details in Section 3.

**Fig 3:**
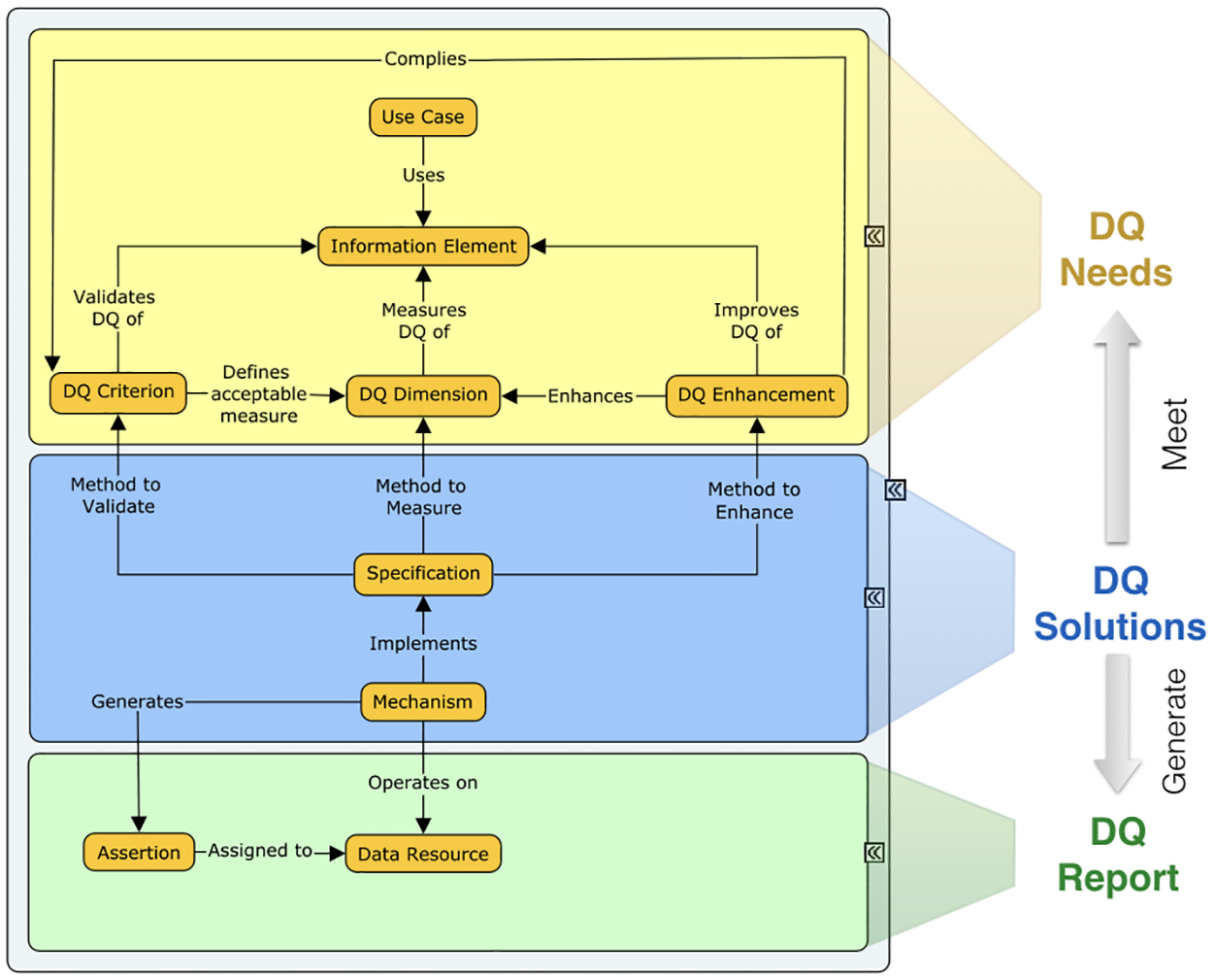
The conceptual framework: Concepts and classes. DQ Needs concepts: Use Case, Information Element, DQ Dimension, DQ Criterion and DQ Enhancement. DQ Solutions concepts: Specification and Mechanism. DQ Report concepts: Data Source and Assertion.

The scenario described demonstrates that three main components are necessary to enable the DQ assessment and management in the context of biodiversity informatics: DQ Needs, DQ Solutions and DQ Report. Accordingly, the proposed conceptual framework defines nine concepts organized in three classes of concepts, as illustrated in Fig 3. In Fig 3, the nine small yellow boxes represent the concepts, which are organized in the three larger boxes that represent the concepts classes.

The next three Sections (2.1, 2.2, 2.3) describe the nine concepts in each respective concepts class. Section 3 illustrates how to use the framework in a practical way. Section 3.1 demonstrates how the framework concepts enable the creation of DQ profiles that represents DQ needs for a specific data purpose. Section 3.2 describes how the concepts can be used to create a DQ status report that enables data users, aggregators, owners, curators and providers to assess and manage the fitness for use of data according to a specific DQ profile.

### 2.1 DQ needs concepts class

The class DQ Needs contains the concepts Use Case, Information Element, DQ Dimension, DQ Criterion and DQ Enhancement. These concepts describe DQ requirements according to the data user’s perspective.

#### 2.1.1 Use Case

By definition, DQ is a concept related to fitness for use [53]. Therefore, it is necessary to clearly define what the data use context is in order to define the meaning of DQ or "fitness for use” in such context. The concept Use Case defines a scope delimitation concerning DQ Needs. A Use Case may represent specific or general task performed by data users with biodiversity data, e.g., to generate a distribution model for the wild bee *Tetragonisca angustula* s.l. in Brazil or to generate species distribution models. In other words, Use Case is a data use story together with its purpose and data transformations/processing.

Every attempt to assess and manage fitness for use should be guided by the Use Case context; that is, it must be used as a reference to define any effort regarding DQ, including actions such as DQ measurement, validation and amendment. Use Cases are inherently framed from the perspective of a data user, but the point of view of other stakeholders, such data curators', may help guide the prioritization of data quality control efforts, given the scarce resources available to biodiversity data providers.

See an example in the Section 1.1 in S1 Appendix for details.

#### 2.1.2 Information Element

An **Information Element (IE)** is an abstraction that represents relevant content in the Use Case context. An IE can be a single element or a set of elements that may represent an event, an object, an abstract data concept such as a GUID (Globally Unique Identifier), or an entity of the real world, and has some importance in a data use context.

IE can be classified as a single IE or a composed IE. For example, "decimal latitude" may be a single IE that represents, in decimal degrees, the position from the Equator to the north (positive values) or to the south (negative values) with valid values between -90 and 90, inclusive, which in biodiversity data is normally found under the Darwin Core term dwc:decimalLatitude. "Decimal coordinates" is an example of a composed IE consisting of decimal latitude, decimal longitude, coordinate uncertainty and geodetic datum, which together represent a position on the surface of the Earth using decimal degrees [54], and are found in biodiversity data under the terms dwc:decimalLatitude, dwc:decimalLongitude, dwc:coordinateUncertantyInMeters, and dwc:geodeticDatum.

Some specific subset of all possible IE is valuable for a specific Use Case. These valuable IE are required and must have an appropriate level of quality to be fit for use in the associated Use Case; therefore, these IE should be the target of efforts concerning DQ, either for quality measurement, validation or amendment. It is essential to identify which IE are valuable for a specific Use Case.

For software implementations, it is also essential to identify which metadata schema and which specific terms are involved in each single or composed IE. To allow software interoperability, it is strongly recommended to define IE based on terms from metadata schema standards of an appropriate domain, such as Darwin Core, Audubon Core, Structured Descriptive Data, Dublin Core, etc. For example, either or both of dwc:decimalLatitude plus dwc:decimalLongitude and dwc:footprintWKT may describe a location in Darwin Core. “A Location may have both a point-radius representation (see dwc:decimalLatitude) and a footprint representation, and they may differ from each other." While in currently available data, dwc:footprintWKT is almost never populated, a Use Case which defines coordinate without reference to dwc:decimalLatitude and dwc:decimalLongitude will leave implementers unclear on how to proceed.

See examples of IE in Section 1.2 in S1 Appendix for details.

#### 2.1.3 DQ Dimension

The representation of an instance of a “thing” (the most general class in an ontology; all individuals are by definition instances of a “thing”, including physical objects, virtual objects, events, collections or any other abstract or concrete elements [55]) is different from the “thing” itself [56]; there is a gap between a “thing" and the data that represent that "thing". This gap can be measured according to different data quality aspects or attributes, called **DQ Dimensions**, such as completeness (measuring the completeness gap between the information and the thing that it represents), precision (measuring the precision gap between the information and the thing that it represents), accuracy, etc. In general, the smaller the gap between a "thing" and the information that represents it, in all the relevant DQ Dimensions in the context of a specific Use Case, the better the quality of the data. For reference, we can list some Dimensions, such as currency, credibility, accuracy, conformity, consistency, integrity, completeness, trust, readability, accessibility, precision and believability [5].

DQ is a multidimensional concept defined by a set of quality attributes [3, 4, 47, 57]. To define DQ in some context, a set of relevant measurable data quality attributes must be determined [48]; that is, the meaning of DQ can be completely and concisely defined by a set of relevant DQ Dimensions for a specific Use Case.

When the quality of some data is measured, a set of Dimensions is used to obtain that quality measurement. For example, data may have high quality for some use when they are complete, precise, credible and accurate for some set of relevant properties. That is, the DQ measurement was high because the measurements of the dimensions of completeness, precision, credibility and accuracy were also high.

The relevance of a single Dimension for a specific use is relative [3, 47] to that use. In a data set that dates from the 1800s with high values on measurements of completeness, precision, credibility, and accuracy, for a use that involves analysis of the current state of the world, the overall DQ could be considered poor because an important dimension for that analysis is the timeliness, and for that data set (accurately representing the state of the world more than 100 years ago), the measurement of timeliness would be low.

There are three approaches for identifying a set of relevant dimensions for a Use Case [1].

- **Intuitive**: based on the researchers’ experience;
- **Theoretical**: based on data deficiencies or DQ problems;
- **Empirical**: based on the data user’s needs.

Despite the extensive number of classical DQ Dimensions discussed in the literature that can be used as reference [5, 56, 58, 59], there is no consensus on what constitutes a good set of DQ Dimensions and on what is an appropriate definition of each Dimension [56]. Therefore any well-defined measurable attribute useful for measuring the quality of data in the context of a Use Case can be adopted as a DQ Dimension.

To measure the quality of data according to a DQ Dimension, it is necessary to contextualize the Dimension, that is to specify a context for the Dimension. The quality of data is measured by a set of abstract Dimensions (e.g. completeness, consistency, conformity) in the context of some specific IE (e.g. coordinates, event date, country, scientific name) and data resource type (i.e. single record or dataset).

For example, the Dimension *completeness* in the context of the IE *coordinates* and the resource type *single record* could measure the presence of values for the latitude, longitude, geodetic datum and coordinate uncertainty in a single record, with a binary conclusion of “is complete” or “is not complete.” Consider, in contrast, the same Dimension of *completeness* still in the context of the IE *coordinates*, but now in the context of the resource type *dataset*. This Dimension in context would measure the proportion of records that have values for latitude, longitude, geodetic datum and coordinate uncertainty, reporting some proportion between zero and one for a given data set. See examples in the Section 1.3 in S1 Appendix for details.

DQ Dimensions are used to measure the quality of single records or datasets, but they are not intended to determine if the quality measures assigned to the data are satisfactory for considering if the data are fit for use in a specific Use Case context. To determine the acceptable measures for the DQ Dimensions for some specific Use Case, it is necessary to go one step further than a Data Quality Dimension in Context and to define DQ Criteria.

#### 2.1.4 DQ Criterion

A **DQ Criterion** is a statement that describes acceptable DQ measures by which data are judged regarding their fitness for some use. DQ Criteria are used to validate whether the quality of data is satisfactory to be used in a specific Use Case. Data compliant with the Criterion means the data are fit for use according to the related DQ Dimension.

For example, "coordinate completeness of a dataset must be equal to 100%” is a Criterion used to validate if the measure of the DQ Dimension completeness, in the context IE coordinates and resource type dataset, has a value equal to 100%. If some data has a measure equaling 100%, then the data are compliant with the Criterion, otherwise, the data are not compliant with the Criterion, and consequently unfit for (this) use. See examples in the Section 1.4 in S1 Appendix for details.

A set of selected Criteria splits data that is fit for use from data which is not fit for use for a particular Use Case.

#### 2.1.5 DQ Enhancement

**DQ Enhancements** are statements that describe activities required to improve DQ. An Enhancement can be a description of a procedure, protocol, training, a best practice or anything that can be used to improve DQ. There are three types of Enhancements:

- **Prevention**: for preventing incidents (errors);
  - Ex.: "Suggest similar and valid scientific names while typing."
- **Correction**: for correcting errors;
  - Ex.: "Fill in taxon hierarchy based on the most specific name."
- **Recommendation**: for recommending corrections.
  - Ex.: "Recommending coordinates based on the locality description."

See examples in the Section 1.4 in S1 Appendix for details. In the domain of biodiversity informatics with distributed data and differing opinions we see prevention and recommendations as more important forms of Enhancements to express than corrections. It will generally be more palatable to a data curator to recommend some change to their data based on some defined criteria than to assert a correction of their data.

### 2.2 DQ solutions concepts class

The concepts Specification and Mechanism belong to the class DQ Solutions. These concepts describe how to proceed and what to use to comply with DQ Needs.

#### 2.2.1 Specification

**Specifications** are a formal or informal description of methods for performing DQ measurements, validations and enhancements. Specifications can be highly mathematical and computational, using any of several formal languages defined for that purpose, or can be informal, using, for example, natural language to describe how to proceed [60]. Preferentially, it is recommended to use both levels of formalization to define a Specification, to make the method description easily readable by both software developer and non-developer audiences (so long as the formal and human readable specifications are and remain consistent).

A measurement of a particular Dimension, a validation according to a specific Criterion or an amendment according to a specific Enhancement could be specified in several different ways. For example, the Dimension "precision" could be measured using a numerical or a statistical approach. The Criterion “Coordinates must be consistent" could specify that data are compliant if “the coordinate falls inside the related country boundary” or if “the coordinate falls inside country boundary or up to 1 km from the boundary of the related country". The Enhancement "Fill taxonomic hierarchy based on the most specific name" could be performed using a number of different taxonomic authority sources and algorithms.

Thus, the concept Specification describes methods, techniques and/or algorithms used to obtain the value of the DQ measurement, validation or enhancement. See examples in the Section 2 in S1 Appendix for details.

#### 2.2.2 Mechanism

A **Mechanism** can be software, hardware, a technique, a tool, a person or any other thing that implements one or more Specifications. Mechanisms generate the measurements, validations and amendment results, collectively called Assertions, according to the methods described by the Specifications.

Critical to understanding the conceptual framework is the distinction between, on one side, Criterion, Dimension and Enhancement, on the other side, and Mechanisms. Criterion, Dimension and Enhancement are statements that describe DQ Needs according to the data user perspective. Mechanisms are technical or nontechnical artifacts used to determine or enforce compliance with the DQ Needs [60].

There are two types of Mechanism based on its coverage:

- **Broad**: implements multiple Specifications.
  - Ex.: DwC-A Validator 2.0 is software that implements several Specifications for asserting DQ validations and enhancements [61].
- **Specific**: implements a single Specification.
  - Ex.: A specific Web Service in the Google Maps Application Programming Interface (API) could be a precise Mechanism designed to implement a specific enhancement Specification, which provides an amendment recommending a value for country name on the basis of provided coordinates.

In some cases, we use more than one Mechanism to implement a Specification. To take advantage of reuse, we can define Mechanisms that depend on (or use) other existing Mechanisms.

For instance, a Specification for measuring the DQ Dimension “Coordinate Conformity of Single Record" requires that the latitude and longitude must be numbers, do not have invalid characters and be in the correct range. In this case, we could implement a Mechanism that uses four other Mechanisms: (1) Check whether each provided value is a number; (2) Check whether each provided value contains only valid characters; (3) Check whether latitude is in the correct range; and (4) Check whether longitude is in the correct range.

Reusing existing Mechanisms wherever possible is strongly encouraged to avoid duplication of effort. See examples in the Section 2 in S1 Appendix for details. Specifications should, by their nature, help facilitate the reuse of existing Mechanisms.

### 2.3 DQ report concepts class

The concepts Data Source and Assertion belong to the class DQ Report. A DQ Report is the target of DQ Solution concepts and is used to support the DQ assessment and management according to the DQ Needs concepts. Informally, users examine DQ reports produced by DQ solutions to assess whether their particular DQ needs are met.

#### 2.3.1 Data Resource

**Data Resource** is an instance of data and the target to the DQ assessment and management.

Data Resources have a property called “resource type.” Resource type, in the context of the conceptual framework, can be "single record" or "multi-record (dataset)". This property is important because it affects the method for measuring, validating and improving a Data Resource. For example, coordinate completeness of a single record could be measured qualitatively by checking whether the latitude and longitude of the record are filled or not; whereas the coordinate completeness of a dataset could be measured quantitatively, measuring the percentage of records in the dataset which have the latitude and longitude fields filled. Both measurements are for coordinate completeness, but they are measured in different ways due to the different resource type.

In the context of this paper, a single record may refer to a database tuple in the strict sense, that is, a row in a concrete table or to a row in a flattened view or query across several related tables. Likewise, structured (e.g. Star Schema, or RDF) DarwinCore representations of an Occurrence (e.g. an Occurrence with a determination history comprising a current identification and five previous identifications), are referred to here as a single record, even though it is composed of several records of structured data. See examples in Section 3 in S1 Appendix for details. Both single record and dataset, as used here, are encoded data with a defined structure, and can be described as dcmitype:Dataset.

#### 2.3.2 DQ Assertion

**A DQ Assertion** is the result of a measurement (of a DQ Dimension), a validation (according to a DQ Criterion) or an amendment (according to a DQ Enhancement), obtained by a Mechanism operating on a specific Data Resource (single record or dataset) using a specific method defined by a Specification. A set of DQ Assertions assigned to a Data Resource defines the current status of quality of the Data Resource for the explicit context of a specific Use Case.

DQ Assertions can be classified into three types: DQ Measures, DQ Validations and DQ Amendments. DQ Measures assign a DQ measure to a Data Resource according to a DQ Dimension, a measurement method defined by a Specification, and the Mechanism used to obtain the measure. DQ Validations assign a DQ validation to a Data Resource according to a DQ Criterion, a validation method, defined by a Specification, and the Mechanism used to obtain the validation. DQ Amendments namely assign a DQ amendment to a specific Data Resource according to a DQ Enhancement, an enhancement method defined by a Specification, and the Mechanism used to obtain the amendment.

Therefore, a DQ Assertion is composed of five components: (1) DQ Dimension or DQ Criterion or DQ Enhancement; (2) Specification; (3) Mechanism; (4) Data Resource and (5) Assertion Result. The component (1) defines the DQ Dimension that was measured, the DQ Criterion on which the validation was based or the DQ Enhancement on which the amendment was based. The component (2) defines which method (Specification) was used to perform the measure, validation or improvement. The component (3) defines the Mechanism used to obtain the assertion. The component (4) defines the specific target of the assertion. Finally, the component (5) defines the asserted result obtained (measure, validation or amendment).

A hypothetical example of a DQ Measure assertion is:

- **DQ Dimension**: Coordinate consistency of single record
- **Specification**: Coordinate is "Consistent" only if the circumference around the position of latitude and longitude, with a radius equal to the coordinate uncertainty in meters, intersects the area within the boundary of the country, according to the Global Administrative Areas [62].
- **Mechanism**: BDQ-Toolkit [63]
- **Data Resource**: decimalLatitude: -35.3848; decimalLongitude: 13.8352; coordinateUncertaintyInMeters: 100; geodeticDatum: SIRGAS2000; countryName: Brazil
- **Assertion result**: Not Consistent

See examples in the Section 3 in S1 Appendix for details.

## 3. Using the conceptual framework

The conceptual framework defines concepts which can be organized to define the meaning of DQ in a specific context, to guide the determination of DQ status of Data Resource to enable the fitness for use assessment and DQ management.

In this context, we show how to use the conceptual framework for DQ profiling (Section 3.1) and DQ status reporting based on a DQ profiling (Section 3.2).

### 3.1 DQ profiling

**Fig 4:**
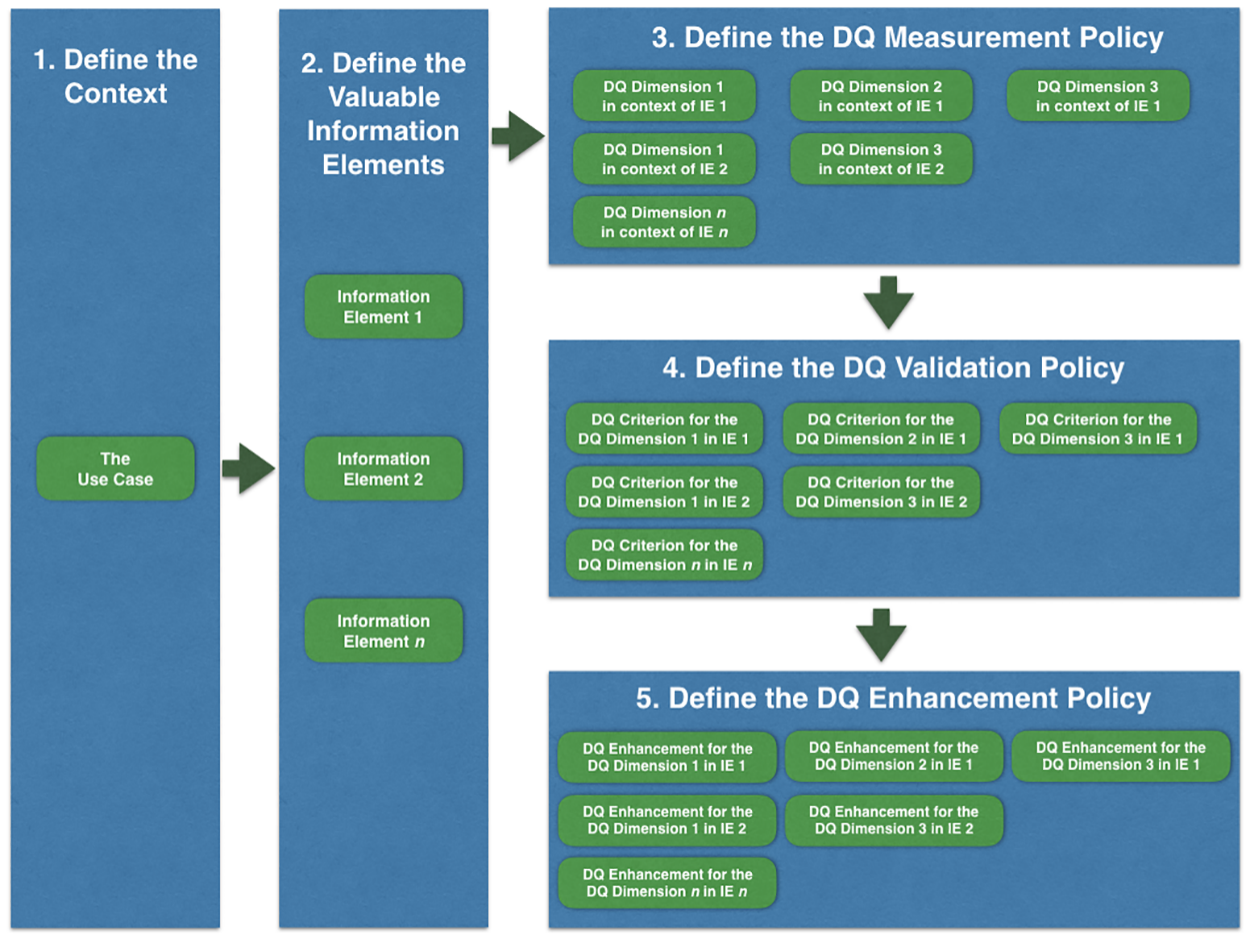
The steps to define a DQ profile. DQ profiling can be performed by the following steps: (1) Define the Context, (2) Define a set of Valuable IE, (3) Define a set of relevant DQ Dimensions as DQ Measurement Policy, (4) Define a set of relevant DQ Criteria as DQ Validation Policy and (5) Define a set of relevant DQ Enhancements as DQ Enhancement Policy.

A **DQ profile** organizes the DQ Needs concepts to clearly describe how DQ must be tackled to enable the DQ assessment and management in a specific Use Case context. The concepts of the DQ Needs class define a set of modular and reusable components for defining DQ requirements. In other words, DQ profile is a formalized “wish-list” for DQ with a certain future use in mind. As illustrated in the Fig 4, to define DQ Needs for a specific Use Case, it is necessary to follow the next five steps:

1. **Define the Context**: describe the Use Case that will be used as a DQ scope delimitation for the next four steps.
2. **Define the Valuable IE**: select and describe a set of IE that are important and should have a satisfactory level of quality for use in the Use Case context.
3. **Define the DQ Measurement Policy**: select and describe a set of DQ Dimensions in context for each relevant Valuable IE, to be measured to assess and manage the fitness for use in the Use Case context. High quality of all the defined contextualized DQ Dimensions indicates that conforming data are highly fit for use in the Use Case context.
4. **Define the DQ Validation Policy**: select and describe a set of DQ Criteria that rule how data must be presented to be considered fit for use in the Use Case context based on the DQ Measurement Policy. Data compliant with all the defined DQ Criteria denote that the data are fit for use in the Use Case context.
5. **Define the DQ Enhancement Policy**: select and describe a set of DQ Enhancements that may make data fitter for use in the Use Case context by enhancing the measures of quality defined by the DQ Measurement Policy and, consequently, may make data compliant with the defined DQ Validation Policy.

The result of these five steps clearly defines a DQ scope delimitation, the information that must have quality in this scope, how to measure the quality of such relevant information, what are the acceptable measures and, finally, how to improve the measures of quality. See an example in Section 1 in S1 Appendix for details.

### 3.2 DQ status reporting

**Fig 5:**
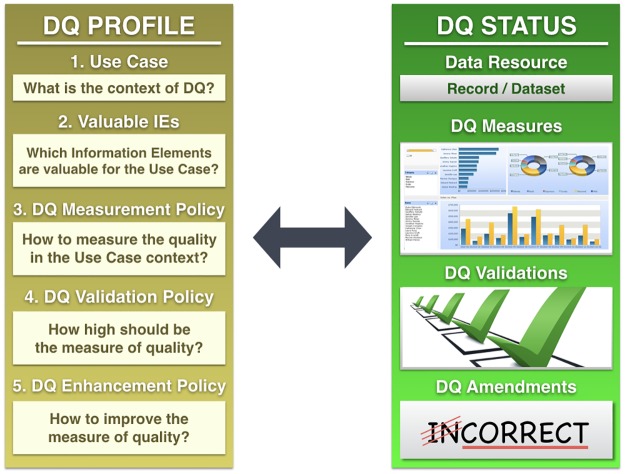
The relation between DQ profile and DQ status. Based on a DQ profile we can report the DQ status of Data Resource in the context of a specific Use Case for each Valuable IE. For each DQ Dimension in the DQ Measurement Policy of the DQ profile, there is a DQ Measure in the DQ status report; for each DQ Criterion in the DQ Validation Policy of the DQ profile, there is a DQ Validation in the DQ status report; and for each DQ Enhancement of the DQ Enhancement Policy of the DQ profile, there is a DQ Amendment (when applicable).

**DQ status** is a subset of DQ Assertions assigned to a specific Data Resource, selected according to a DQ profile. The goal of the DQ status is to enable the DQ assessment and management of a Data Resource according to a particular DQ profile, as illustrated in Fig 5.

A DQ status report describes the current and relevant level of quality of a specific Data Resource according to a particular DQ profile. For each Data Resource, a DQ report presents three components: (1) DQ Measures—a set of DQ Measures assertions of the DQ Dimensions defined by the DQ Measurement Policy of a DQ profile; (2) DQ Validations—a set of DQ Validations assertions according to the DQ Criteria defined by the DQ Validations Policy of a DQ profile; (3) DQ Amendments—a set of DQ Amendments assertions according to the DQ Enhancements defined by the DQ Enhancement Policy of a DQ profile. DQ Amendments asserting that changes should be made to the original data are best phrased as “proposed,” to make it clear that their acceptance is a decision of the data curators or data owners. See examples in Section 3 in S1 Appendix for details of DQ reporting according to the DQ profile presented in Section 1 in S1 Appendix.

A DQ status can clearly report the relevant components of quality about a Data Resource for enabling the DQ assessment and management.

## 4. Discussion

A consistent approach to assess and manage DQ is critical for biodiversity data users [46]. However, achieving this goal has been particularly challenging because of the idiosyncrasies inherent to the concept of quality [64]. DQ assessment and management cannot be performed if we have not clearly established the quality needs of a data user [65].

The proposed conceptual framework supports the biodiversity informatics community to describe the meaning of "fitness for use" from a data user’s perspective in a common and standardized way. Based on well-established principles and concepts from DQ literature [1], the framework allows the biodiversity informatics community to organize the way it addresses DQ so that data users can judge whether data are fit for use for a particular purpose and data owners can improve the quality of unsuitable data.

**Fig 6:**
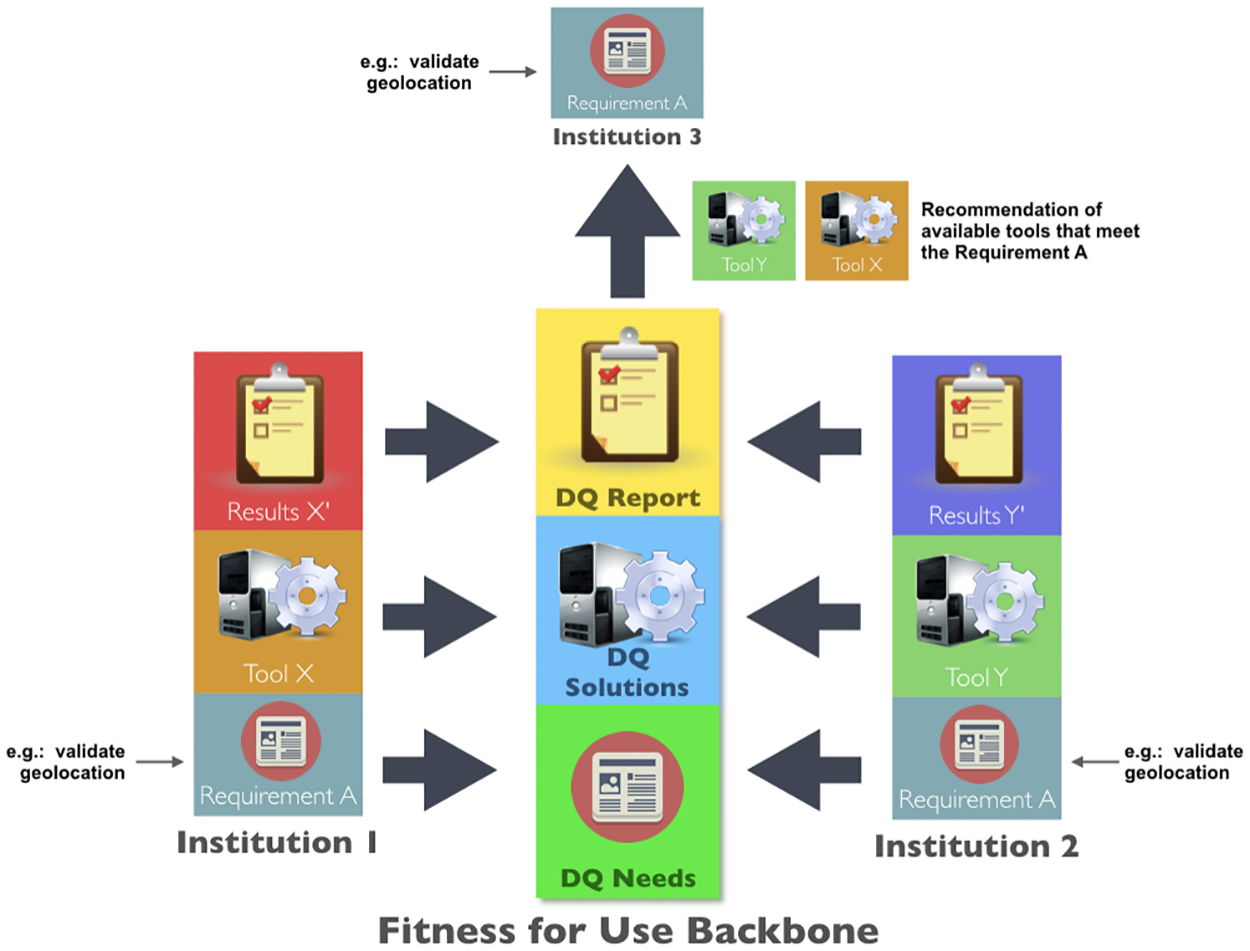
A Fitness for Use Backbone. Basic architecture for a computational platform based on the conceptual framework, composed of three main parts: (1) DQ Report (registering and retrieving DQ status assigned to Data Resources according to DQ profiles definitions), (2) DQ Solutions (registering and retrieving methods and tools for meeting DQ needs) and (3) DQ Needs (registering and retrieving entire DQ profiles or individual parts, such DQ Dimensions, DQ Criterion and DQ Enhancement).

With a common "Fitness For Use Backbone" (FFUB) backed by the conceptual framework, the biodiversity informatics community would have a tool to express and share its understanding of DQ needs and DQ solutions in a common way, thus improving the synergy of the community and consequently increasing the reusability of solutions and decreasing the duplication of efforts, as illustrated in the Fig 6.

For example, if an institution (as Institution 1, in Fig 6) has a specific requirement (e.g. validate geolocation), it can implement a tool (as Tool X, in Fig 6) to meet such requirements, that will generate results (as Results X’, in Fig 6). Concurrently, another different institution (as Institution 2, in Fig 6) may have the same requirement (i.e. validate geolocation) and it also implements a tool (as Tool Y, in Fig 6) to meet the requirement that also will generate results (as Results Y’, in Fig 6). This scenario clearly demonstrates duplication of effort when two different tools are developed to meet the same requirement. Also, the results generated by the tools, usually can't be compared, and may be inconsistent, because they do not share a common standard.

If all the requirements, tools and results were mapped to a common standard, it would enable interoperability among the metadata about such components, making them comparable, sharable and reusable. It would enable, for example, an institution (as Institution 3, in Fig 6), which has a DQ requirement (e.g. validate geolocation) but doesn’t have a tool to meet such requirement, to search in this “common Fitness for Use Backbone" for available tools being used by other institutions to meet the specific requirement. In this scenario, the conceptual framework can map the requirements, tools and results by respectively using the concepts of DQ Needs, DQ Solutions and DQ Report classes.

With the implementation of the collaborative platform promoted by the framework, we can generate a searchable repository of common and reusable DQ profiles, DQ reports, Use Cases, IE, DQ Dimensions, DQ Criteria, DQ Enhancements, Specifications and Mechanisms for a range of data uses, enabling institutions to compose their DQ profiles to better suit their goals by reusing the DQ Needs and DQ Solutions shared by other institutions.

**Fig 7:**
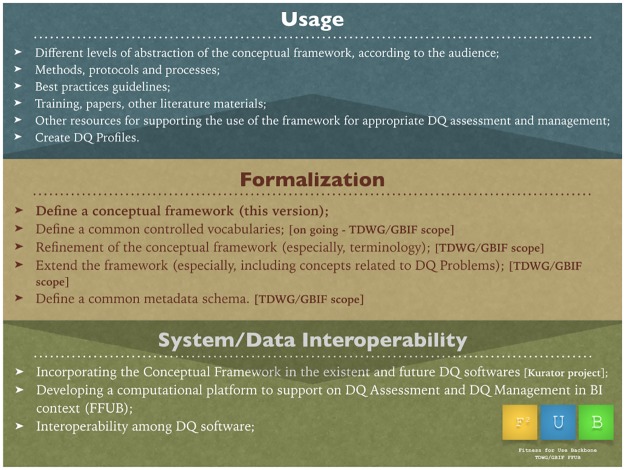
Roadmap for implementing the conceptual framework. The roadmap is organized into three levels: Usage (to bring the conceptual framework closer to the reality of different stakeholders, by presenting it with different abstractions depending the audience), Formalization (to continue the progress of this work, going deeper on the formalization and extending the current version of the conceptual framework) and System/Data Interoperability (to enable the interoperability of DQ related systems and data).

To achieve a level of implementation of the conceptual framework, however, as illustrated in Fig 6, in a way that allows computational interoperability and a broader adoption, it is necessary to follow a roadmap, as illustrated by Fig 7, which is organized in three levels: usage, formalization and system/data interoperability.

The presented framework is in a formalization level, where we define the concepts to describe the necessary components to deal with DQ Needs, DQ Solutions and DQ Reports to enable DQ assessment and management. Still, in the formalization level, we are working on a controlled vocabulary for some of the framework concepts. We also plan to discuss and refine the terminology of the framework, extend it to include new concepts and define a common metadata schema.

For example, the authors have considered, at length, the word to use to express the concept DQ Amendment, with enhancement, improvement, and amendment all being candidates. We adopt amendment as it most readily carries the connotation of a proposal for a change rather than a bald assertion. The language proposed amendment, rejected amendment, and accepted amendment comes naturally without connotations of value. Thus the fundamental concept Enhancement relates to derived concepts Enhancement in Context, Enhancement Policy in data quality needs, Enhancement Method in data quality solutions, but to DQ Amendment in DQ reports.

Primarily, those actions will be undertaken in the context of the TDWG/GBIF community, but we can potentially extend it to collaboration with the Assessment of Data Fitness Use Working Group [66] of the Research Data Alliance (RDA), since this initiative has been working on a similar approach to this work, and both initiatives may take advantage of this collaboration.

With a proper formalization, we will be able to work at a system/data interoperability level, which includes incorporating the framework into existing and future DQ software, such as that already being done in the Kurator project, developing the mentioned FFUB and enable interoperability among different DQ software. We are working with partners such as Museum of Comparative Zoology of Harvard University, GBIF Secretariat, iDigBio, Brazilian Biodiversity Information System (SiBBr), Atlas of Living Australia (ALA), VertNet, Ocean Biogeographic Information System (OBIS) and other potential collaborators to further this aim.

We also plan to foster the use of the conceptual framework in the TDWG/GBIF community by presenting material about how the framework can be useful for stakeholders from different biodiversity communities and disciplines.

Another strength of the presented conceptual framework is the division of the concepts into three classes, which increases the potential for mutual collaboration between stakeholders with different skills and knowledge. Biodiversity experts typically are better able to address concepts belonging to the DQ Needs class, whereas concepts belonging to DQ Solutions are typically better addressed by informatics experts. With this separation, data users can individually define DQ needs, and developers can propose already existing DQ solutions for attending them or can develop new mechanisms when necessary. Also, this model allows us to systematically identify gaps on DQ solutions, for example, to identify that there is no effective DQ measurement method for measuring a determined DQ Dimension or that there is a measurement method, but there is no Mechanism that implements it.

The DQ Report concepts class allows the generation of comparable DQ documentation that can be interpreted by data users to judge whether a Data Resource is fit for use and how its quality can be improved. When data users or data owners have DQ report elements (DQ Measures, Validations, and Amendments) assigned to a Data Resource, they can make a coherent assessment concerning the fitness for the use of the Data Resource and compare its quality with that of other Data Resources.

Improvement in biodiversity data is an essential step for many workflows in biodiversity sciences, located between data generation and aggregation and the statistical analysis of improved data. Research satisfaction and trust in the results is often based on loose feelings or strict measurements of the data to be “enough to answer the research questions.” Understanding “what works” and “how much is enough” can be supported by the presented conceptual framework.

The conceptual framework was designed specifically for tackling DQ issues in the biodiversity informatics domain. However, the presented conceptual framework can potentially be applied without adaptation to any other domain because the concepts are generic enough to be domain independent. The generality of the framework enables its use for others domains, especially those scenarios which share some specific features, as previously mentioned, *viz*.: deal with distributed data, have a diverse community of users and a wide range of data usages, where there are competing concepts (e.g. taxonomies, [49]) and the DQ requirements cannot always be clearly and easily defined and universally accepted.

## 5. Final remarks

The conceptual framework presented here is the foundation for DQ Task Group 1 of the Biodiversity Data Quality Interest Group (BDQ-IG) [67], created by TDWG and merged with an ongoing discussion group of GBIF on Biodiversity Data Quality. The goal of this task group is to develop a conceptual framework that serves as a common ground for a collaborative development of solutions (encompassing tools, policies, and concepts) for DQ assessment and management based on data fitness for use [68].

The BDQ-IG has created two additional task groups within TDWG. Task Group 2 addresses DQ Tools Tests and Assertions, to catalog existing DQ tools, tests and assertions and to identify gaps concerning the availability of these, and Task Group 3 addresses DQ Use Cases, to identify DQ requirements for specific uses of data. In this context, the presented Framework can be used to map the outcomes from the Use Cases Task Group into the DQ Needs concepts, based on data users’ expertise/knowledge and to map the outcomes from the DQ Tools Task Group into DQ Solutions concepts based on developers’ expertise. In this way, all groups can work in parallel, while maintaining the same foundation.

Also, the presented conceptual framework has been used to support two GBIF task groups formed of experts on Species Distribution Modeling and Agrobiodiversity, convened by the GBIF Secretariat, that have been working to shape the meaning of data fitness for use in the context of Species Distribution Modeling and Agrobiodiversity.

As further work, the Task Group 1 of the TDWG/GBIF BDQ IG has established an agenda to foster the use of the conceptual framework as common ground to tackle DQ issues among the community. This agenda involves presenting the formal conceptual framework in a more practical way to bring it closer to the reality of different stakeholders, such as data providers, data and collections curators and holders, digitizers, taxonomists, software programmers and data users. This will include practical guidelines, training material and interactive tools for DQ profiling and reporting.

## Supporting information

S1 Appendix Framework validation—Proof of concept. The purpose of this document is to present a proof of concept that tests all the concepts of the framework and shows how it can be used to assess and manage the fitness for use of biodiversity data, providing a worked example with a real dataset.
(PDF)

S2 Appendix Glossary of terms. The purpose of this document is to serve as quick reference for definitions of terms frequently used in this work.
(PDF)

## References

[1] Ge M, Helfert M. A review of information quality research-develop a research agenda. ICIQ Paper, MIT; 2007. pp. 76–91.

[2] Berndt DJ, Fisher JW, Hevner AR, Studnicki J. Healthcare Data Warehousing and Quality Assurance, Computer; 2001; pp. 56–65.

[3] Chapman AD. Principles of data Quality. Report for the Global Biodiversity Information Facility; 2005, [cited 15 Mar 2015] pp. 61. Copenhagen: Global Biodiversity Information Facility. http://www.gbif.org/orc/?doc\_id=1229.

[4] Strong DM, Lee YW, Wang RY. Data Quality in context. Commun ACM. 1997;40: 103–110.

[5] Cai L, Zhu Y. The Challenges of Data Quality and Data Quality Assessment in the Big Data Era. Data Science Journal; 2015; 14(0), p.2 http://doi.org/10.5334/dsj-2015-002

[6] Johnson J, Leitch R, Neter J. Characteristics of Errors in Accounts Receivable and Invetory Audits. Accounting Review. 1981; 56 (2): 270–293.

[7] Redman T. Data Quality: The Field Guide. DigitalPress; 2001; ISBN:1555582516.

[8] Huang KT, Lee YW, Wang RY. Quality Information and Knowledge Management. Prentice Hall 1st ed; 1999; ISBN: 0130101419

[9] Lee Y, Strong D, Kahn B, Wang RY. AIMQ: A Methodology for Information Quality Assessment, Information & Management; 2002; Volume 40, Issue 2, pp. 133–146.

[10] Pipino L, Lee YW, Wang RY. Data Quality Assessment, Communications of the ACM; 2002; pp. 211–218.

[11] Stvilia B, Gasser L, Twidale MB, Smith LC. A Framework for Information Quality Assessment, Journal of the American Society for Information Science and Technology; 2006.

[12] Wang RY. A Product Perspective on Total Data Quality Management, Communications of the ACM; 1998; pp. 58–63.

[13] Eppler MJ. Managing Information Quality. Springer, 2nd ed; 2006; ISBN:978-3-540-31408-0

[14] Chengalur-Smith IN, Ballou DP, Pazer HL. The Impact of Data Quality Information on Decision Making: An Exploratory Analysis. IEEE Trans. Knowl. Data Eng; 1999; 11(6): 853–864

[15] Teflian M. Information Liquidity. Time0/PerotSystems Cambridge, MA 1999.

[16] Kaplan D, Krishnan R, Padman R, Peters J. Assessing Data Quality in Accounting Information Systems, Communications of the ACM. 2;1998; pp. 72–78.

[17] Busby JR. Australian Biotaxonomic information system. Introduction and data interchange standards. Canberra: Australian Biological Resources Study, Department of Science and the Environment; 1979 pp. 25.

[18] ASC [Association of Systematics Collections]. An information model for biological collections. Report of the Biological Collections Data Standards Workshop, 8–24 August 1992. ASC. Washington DC [cited 2015 Mar 10] http://cool.conservation-us.org/lex/datamodl.html

[19] Croft JR, editor. HISPID—herbarium information standards and protocols for interchange of data. Canberra: Australian National Botanic Gardens; 1989.

[20] Conn BJ, editor. HISPID3. Herbarium information standards and protocols for interchange of data. Version 3; 1996 [cited 2015 Aug 2015]. Sydney: Royal Botanic Gardens, http://www.rbgsyd.gov.au/HISCOM.

[21] TDWG [Biodiversity Information Standards]. HISPID3 –herbarium information standards and protocols for the interchange of data; 2007 [cited 2015 Mar 10] http://www.tdwg.org/standards/110.

[22] Stockwell D, Peters D. The GARP modelling system: problems and solutions to automated spatial prediction. Int J Geogr Inf Sci. 1999;13: 143–158.

[23] Phillips SJ, Anderson RP, Schapire RE. Maximum entropy modeling of species geographic distributions in proceedings 21st International Conference on Machine Learning; 2004 [cited 2015 Mar 10]. pp. 655–622. http://www.cs.princeton.edu/~schapire/papers/maxent_icml.pdf.

[24] Edwards JL, Lane MA, Nielsen ES. Interoperability of biodiversity databases: biodiversity information on every desktop. Science. 2000;289: 2312–2314. 11009409 10.1126/science.289.5488.2312

[25] Sunderland ME. Computerizing Natural History Collections. Endeavour; 2013; 37(3): 150–161. doi: 10.1016/j.endeavour.2013.04.001 23664113 10.1016/j.endeavour.2013.04.001

[26] Rosenberg G. A database approach to studies of molluscan taxonomy, biogeography and diversity, with examples from Western Atlantic marine gastropods. American Malacological Bulletin; 1993 10:257–266

[27] Williams SM., Smolen MJ., Brigda AA. Documentation standards for automatic data processing in Mammalogy. The Museum of Texas Tech University 1979; vii pp. 48 Lubbock, TX.

[28] MVZ [Museum of Vertebrate Zoology, Berkeley]. The MVZ Collections Information Model. 1996. [cited 2015 Mar 10] http://mvz.berkeley.edu/cis/mvzmodel.pdf

[29] Chapman AD. Australian plant name index Australian Flora and fauna series, series Nos 12–15. Canberra: Australian Government Publishing; 1991 pp. 1–3053.

[30] ABRS. Australian faunal directory; 2004 Canberra: Australian Biological Resources Study [cited 2015 Aug 21] http://www.environment.gov.au/biodiversity/abrs/online-resources/fauna/index.html.

[31] Missouri Botanical Garden. W3 TROPICOS; 2001 [cited 2015 Mar 15]. http://mobot.mobot.org/W3T/Search/vast.html

[32] IPNI. International plant names index; 1999 [cited 2015 Mar 15]. http://www.ipni.org/index.html.

[33] TDWG [Biodiversity Information Standards]. About TDWG; 2015. [cited 2015 Mar 10] http://www.tdwg.org/about-tdwg/.

[34] Yesson C, Brewer PW, Sutton T, Caithness N, Pahwa JS, Burgess M, et al How Global Is the Global Biodiversity Information Facility? Beach J, editor. PLoS One [Internet]. Public Library of Science; 2007 11 7 [cited 2017 Jan 26];2(11):e1124 Available from: http://dx.plos.org/10.1371/journal.pone.0001124 10.1371/journal.pone.0001124 PMC2043490 17987112

[35] GBIF [Global Biodiversity Information Facility]. What is GBIF; 2015. [cited 2015 Mar 10] http://www.gbif.org/what-is-gbif.

[36] Chapman AD. Quality control and validation of environmental resource data in data quality and standards: proceedings of a seminar organized by the Commonwealth Land Information Forum, Canberra; 5 December 1991. Canberra: Commonwealth Land Information Forum, 1992.

[37] Chapman AD. Environmental data quality–b. Data cleaning tools. In: Appendix I to Sistema de Informação Distribuído para Coleções Biológicas: a Integração do Species Analyst e SinBiota. FAPESP/Biota Process no. 2001/02175-5 March 2003–March 2004; [cited 2015 Jun 28]. 57 pp. São Paulo Research Foundation, Campinas, Brazil: CRIA 2004. http://splink.cria.org.br/docs/appendix_i.pdf.

[38] Chapman AD. Principles and methods of data cleaning primary species occurrence data. Report for the Global Biodiversity Information Facility; 2005 [cited 2015 Mar 15] pp. 75. Copenhagen: Global Biodiversity Information Facility. http://www.gbif.org/orc/?doc\_id=1262.

[39] Dalcin EC. Data quality concepts and techniques applied to taxonomic databases. PhD Dissertation. University of Southampton, UK. 2005.

[40] Veiga AK, Saraiva AM, Cartolano EA. Data quality concepts and methods applied to biological species occurrence data In: ICT for agriculture, rural development and environment–where we are? Where we will go? Prague: Czech Centre for Science and Society; 2012.

[41] Dou L, Cao G, Morris PJ, Morris RA, Ludäscher B, Macklin JA, et al Kurator: a Kepler package for data curation workflows. Procedia Comput Sci. 2012;9: 1614–1619.

[42] Veiga AK, Cartolano EA, Saraiva AM. Data Quality control in biodiversity informatics: the case of species occurrence data. IEEE Latin America Transactions. 2014;12: 683–693.

[43] Otegui J, Ariño AH, Encinas MA, Pando F. Assessing the Primary Data Hosted by the Spanish Node of the Global Biodiversity Information Facility (GBIF). PLoS ONE 8(1): e55144 2013; doi: 10.1371/journal.pone.0055144 23372828 10.1371/journal.pone.0055144 PMC3555939

[44] Otegui J, Guralnick RP. The geospatial data quality REST API for primary biodiversity data. Bioinformatics. 2016: btw057v2–btw057. http://bioinformatics.oxfordjournals.org/content/early/2016/02/16/bioinformatics.btw057.full 10.1093/bioinformatics/btw057 PMC4892415 26833340

[45] Beck J, Böller M, Erhardt A, Schwanghart W. Spatial bias in the GBIF database and its effect on modeling species’ geographic distributions. Ecol Inform. 2014;19: 10–15.

[46] Maldonado C, Molina CI, Zizka A, Persson C, Taylor CM, Albán J, et al Estimating species diversity and distribution in the era of big data: to what extent can we trust public databases? Glob Ecol Biogeogr. 2015;24: 973–984. doi: 10.1111/geb.12326 27656106 10.1111/geb.12326 PMC5012125

[47] McGilvray D. Executing data quality projects: ten steps to quality data and trusted information. Morgan Kaufmann Publishing House, Elsevier; 2008.

[48] Wang RY, Strong DM. Beyond accuracy: what data quality means to data consumers. J Manag Inf Syst. 1996;12: 5–33.

[49] Nelson D. Phage taxonomy: we agree to disagree. J Bacteriol [Internet]. American Society for Microbiology; 2004 11; 186(21):7029–31. [cited 2017 Jan 26] Available from: http://www.ncbi.nlm.nih.gov/pubmed/15489416 10.1128/JB.186.21.7029-7031.2004 PMC523225 15489416

[50] Wang YR, Ziad M, Lee YW. Data quality. Kluwer Academic Publishers; 2001 167 p. ISBN 0792372158.

[51] SearchCIO. What is Total Quality Management (TQM)? [Internet]. TechTarget 2005 [cited 2016 Aug 30]. http://searchcio.techtarget.com/definition/Total-Quality-Management

[52] Lee YW. Journey to data quality. MIT Press; 2006 Cambridge, MA.

[53] Chrisman NR. The role of quality information in the long-term functioning of a GIS. APRS. 1983;2: 303–321.

[54] Chapman AD, Wieczorek J, editors. Guide to best practices for georeferencing. Copenhagen: Global Biodiversity Information Facility; 2006.

[55] Noy NF, McGuinness DL. What is an ontology and why we need it. Stanford University, Stanford, CA, 94305. [cited 2016 May 31]. http://protege.stanford.edu/publications/ontology_development/ontology101-noy-mcguinness.html

[56] Wand Y, Wang RY. Anchoring data quality dimensions in ontological foundations. Commun ACM. 1996;39: 11:86–95.

[57] Wang RY, Reddy MP, Kon HB. Toward quality data: an attribute- based approach. Decision Support Systems. 1995;13: 349–372.

[58] Askham N, Cook D, Doyle M, Fereday H, Gibson M, Landbeck U, et al The six primary dimension for data quality assessment-defining data quality dimensions. DAMA; 2013; [cited 2015 Aug 21] http://www.damauk.org/RWFilePub.php?&cat=403&dx=2&ob=3&rpn=catviewleafpublic403&id=106193.

[59] Fox C, Levitin A, Redman T. The notion of data and its quality dimensions. Inf Process Manag. 1994;30: 9–19.

[60] Bishop M. Computer security: art and science. Section 1.5.1. Addison-Wesley; 2003 Boston, MA, USA.

[61] GBIF [Global Biodiversity Information Facility]. dwca-validator, GitHub repository; 2015 [cited 2015 June 14]. Accessed: https://github.com/gbif/dwca-validator.

[62] GADM [Global Administrative Areas]. GADM database of Global Administrative Areas. [cited 2016 May 31]. http://www.gadm.org/

[63] BDQ Toolkit. bdq-toolkit-case, GitHub repository; 2017 [cited 2017 January 19]. Accessed: https://github.com/allankv/bdq-toolkit-case.

[64] Klobas JE. Beyond information quality: fitness for purpose and electronic information resource use. J Inf Sci. 1995;21: 95–114.

[65] Nicolaou AI, McKnight DH. Perceived information quality in data exchanges: effects on risk, trust, and intention to use. Inf Syst Res. 2006;17: 332–351.

[66] Research Data Alliance [RDA]. Assessment of Data Fitness for Use RDA/WDS WG Case Statement [Internet]. 2016. [cited 2015 Aug 21] https://www.rd-alliance.org/group/assessment-data-fitness-use/case-statement/assessment-data-fitness-use-wg-case-statement

[67] BDQIG. GBIF Community Site [Internet]: GBIF/TDWG biodiversity data quality interest group; 2015. [cited 2015 Aug 21] http://community.gbif.org/pg/groups/21292/gbiftdwg-biodiversity-data-quality-interest-group/.

[68] TDWG [Biodiversity Information Standards]. Task Group Charter: BDQ Framework. 2015. [cited 2016 May 19] http://www.tdwg.org/activities/biodiversity-data-quality/bdq-framework/task-group-charter/

