# Supplementary material for: A conceptual framework for quality assessment and management of biodiversity data
Source: PLoS One. 2017 Jun 28;12(6):e0178731. doi: 10.1371/journal.pone.0178731 (PMC5489162; doi:10.1371/journal.pone.0178731)
Supplement: S1 Appendix — The purpose of this document is to present a proof of concept that tests all the concepts of the framework and shows how it can be used to assess and manage the fitness for use of biodiversity data, providing a worked example with a real dataset. (PDF) [file pone.0178731.s001.pdf]

Conceptual Framework on Biodiversity Data Quality

S1 Supporting Information: Framework Validation

# - Proof of Concept -

F² U B

Authors: Allan Koch Veiga, Arthur David Chapman, Paul John Morris, Christian Gendreau, Dmitry Schigel, Tim James Robertson, Antonio Mauro Saraiva

The purpose of this document is to present a proof of concept that allow to stress all the concepts of the framework, and show how it can be used to assess the fitness for use of biodiversity data. This document does not intend to present a complete reference for dealing with DQ, but illustrate with simple examples how to proceed to instantiate all the concepts of the framework. This proof of concept was conceived based on a case study conducted in the Museum of Comparative Zoology of Harvard University using a Dataset from Arizona State University Hasbrouck Insect Collection (http://symbiota4.acis.ufl.edu/scan/portal/collections). All data used and generated in the study case are available at: http://case.bdq.biocomp.org.br:3010/explorer.

This document is organized into three sections: DQ profile, DQ solutions and DQ status. The DQ profile section define the DQ needs for a specific Use Case; the DQ solutions defines the solutions used to meet the DQ needs described in the DQ profile; and the DQ status reports the status of quality of two different Data Resources based on the DQ profile.

1. DQ Profile

1.1. Use Case

Curation of Biological Collections: This Use Case is related to the curation tasks required to keep biological collections data fit for general uses, such as basic geospatial visualization and analysis where data of location, date and taxon of species occurrences are required.

1.2. Valuable Information Elements

List of Information Elements that are particularly important for being used in the Use Case. Those Information Elements must have suitable level of quality for being considered fit for use in the Use Case context.

Coordinates

Information Element description	DwC Elements
The geographic coordinates (in decimal degrees, using the spatial reference system given in geodeticDatum) of the geographic center of a species occurrence.	dwc:decimalLatitude, dwc:decimalLongitude, dwc:geodeticDatum

Scientific name

Information Element description	DwC Elements
The scientific name of the species (typically genus + specific epithet) and authorship.	dwc:scientificName, dwc:scientificNameAuthorship

Collected date

Information Element description	DwC Elements
The date when the collection event was performed.	dwc:eventDate

OCCURRENCE

Information Element description	DwC Elements
An occurrence comprises the location and date where a species sample was collected or observed and its identification with its scientific name.	dwc:decimalLatitude, dwc:decimalLongitude, dwc:geodeticDatum, dwc:scientificName, dwc:scientificNameAuthorship, dwc:eventDate

1.3. DQ Measurement Policy

List of Dimensions in context that are particularly important for supporting the assessment of the fitness for use in the Use Case context. High quality of those Dimensions in context denote that data are pretty fit for use in the defined Use Case. Those Dimensions in context will be the target of the DQ Amendment.

Coordinates completeness of single records

Measure the presence of value for all elements of the Coordinates. The measure will be “COMPLETE" when latitude, longitude and *geodetic datum* values was supplied, else the measure will be “NOT COMPLETE".

DQ Dimension	Information Element	Resource Type
Completeness	Coordinates	Single Record

COORDINATES CONSISTENCY OF SINGLE RECORDS

Measure the consistency of the Coordinates with the associated country, state/province, county and locality of the record.

DQ Dimension	Information Element	Resource Type
Consistency	Coordinates	Single Record

SCIENTIFIC NAME COMPLETENESS OF SINGLE RECORDS

Measure the presence of value for all elements of the Scientific Name. The measure will be “COMPLETE" when scientific name and scientific name authorship values was supplied, else the measure will be “NOT COMPLETE".

DQ Dimension	Information Element	Resource Type
Completeness	Scientific Name	Single Record

SCIENTIFIC NAME CONFORMITY OF SINGLE RECORDS

Measure the conformity of the Scientific Name values with nomenclature authorities.

DQ Dimension	Information Element	Resource Type

Conformity	Scientific Name	Single Record

COLLECTED DATE COMPLETENESS OF SINGLE RECORDS

Measure the presence of value for the Collected Date. The measure will be "COMPLETE" when event date value was supplied, else the measure will be "NOT COMPLETE".

DQ Dimension	Information Element	Resource Type
Completeness	Collected Date	Single Record

COLLECTED DATE CONSISTENCY OF SINGLE RECORDS

Measure the consistency between the Collected Date and the modified date and the life span of the collector.

DQ Dimension	Information Element	Resource Type
Completeness	Collected Date	Single Record

OCCURRENCE COMPLETENESS OF SINGLE RECORDS

Measure the presence of value for all elements of the Scientific Name. The measure will be "COMPLETE" when latitude, longitude, *datum*, scientific name, scientific name authorship and event date values was supplied, else the measure will be "NOT COMPLETE".

DQ Dimension	Information Element	Resource Type
Completeness	Occurrence	Single Record

OCCURRENCE ACCURACY OF SINGLE RECORDS

Measure the accuracy of the Occurrence based on the consistency of Coordinates, the conformity of the Scientific Name and the consistency of the Collected Date.

DQ Dimension	Information Element	Resource Type
Accuracy	Occurrence	Single Record

COORDINATES COMPLETENESS OF MULTI-RECORDS

Measure the proportion of records that have value for all the elements of the Coordinates.

DQ Dimension	Information Element	Resource Type
Completeness	Coordinates	Multi-Record

COORDINATES CONSISTENCY OF MULTI-RECORDS

Measure the proportion of records where the Coordinates is consistent with the associated country, state/province, county and locality.

DQ Dimension	Information Element	Resource Type
Consistency	Coordinates	Multi-Record

SCIENTIFIC NAME COMPLETENESS OF MULTI-RECORDS

Measure the proportion of records that have value for all the elements of the Scientific Name.

DQ Dimension	Information Element	Resource Type
Completeness	Scientific Name	Multi-Record

SCIENTIFIC NAME CONFORMITY OF MULTI-RECORDS

Measure the proportion of records where the Scientific Name values is conform with nomenclature authorities.

DQ Dimension	Information Element	Resource Type
Conformity	Scientific Name	Multi-Record

COLLECTED DATE COMPLETENESS OF MULTI-RECORDS

Measure the proportion of records that have value for the Collected Date element.

DQ Dimension	Information Element	Resource Type
Completeness	Collected Date	Multi-Record

COLLECTED DATE CONSISTENCY OF MULTI-RECORDS

Measure the proportion of records where the Collected Date is consistent with the modified date and the life span of the collector.

DQ Dimension	Information Element	Resource Type
Consistency	Collected Date	Multi-Record

OCCURRENCE COMPLETENESS OF MULTI-RECORDS

Measure the proportion of records that have value for all the elements of the Occurrence.

DQ Dimension	Information Element	Resource Type
Completeness	Occurrence	Multi-Record

OCCURRENCE ACCURACY OF MULTI-RECORDS

Measure the proportion of records where the Coordinates is consistent, the Scientific Name is conforms with nomenclatural authorities and the Collected Date is consistent.

DQ Dimension	Information Element	Resource Type
Accuracy	Occurrence	Multi-Record

1.4. DQ Validation Policy

List of Criteria that are particularly important to rule how data must be presented to be considered fit for use in the Use Case context. Data compliant with these Criteria are fit for use in the defined Use Case context. Making the data compliant with these Criteria will be the target of the DQ Amendment.

Coordinates must be complete

Data with measure of "coordinates completeness of single records" equal "COMPLETE" are COMPLIANT with this Criterion.

Related Dimension in Context	Information Element	Resource Type
Coordinates Completeness of Single Records	Coordinates	Single Record

Coordinates must be consistent

Data with measure of "coordinates consistency of single records" equal "CONSISTENT" are COMPLIANT with this Criterion.

Related Dimension in Context	Information Element	Resource Type
Coordinates Consistency of Single Records	Coordinates	Single Record

scientific name must be complete

Data with measure of "scientific name completeness of single records" equal "COMPLETE" are COMPLIANT with this Criterion.

Related Dimension in Context	Information Element	Resource Type
Scientific Name Completeness of Single Records	Scientific Name	Single Record

Scientific name must be conform with nomenclatural authorities

Data with measure of "scientific name conformity of single records" equal "CONFORM" are COMPLIANT with this Criterion.

Related Dimension in Context	Information Element	Resource Type
Scientific Name Conformity of Single Records	Scientific Name	Single Record

Collected date must be complete

Data with measure of "collected date completeness of single records" equal "COMPLETE" are COMPLIANT with this Criterion.

Related Dimension in Context	Information Element	Resource Type
Collected Data Completeness of Single Records	Scientific Name	Single Record

COLLECTED DATE MUST BE CONSISTENT

Data with measure of "collected date consistency of single record" equal "CONSISTENT" are COMPLIANT with this Criterion.

Related Dimension in Context	Information Element	Resource Type
Collected Data Consistency of Single Records	Collected Date	Single Record

OCCURRENCE MUST BE COMPLETE

Data with measure of "occurrence completeness of single records" equal "COMPLETE" are COMPLIANT with this Criterion.

Related Dimension in Context	Information Element	Resource Type
Occurrence Completeness of Single Records	Occurrence	Single Record

OCCURRENCE MUST BE ACCURATE

Data with measure of "occurrence accuracy of single record" equal "ACCURATE" are COMPLIANT with this Criterion.

Related Dimension in Context	Information Element	Resource Type
Occurrence Accuracy of Single Records	Occurrence	Single Record

ALL THE MULTI-RECORD RECORDS MUST HAVE COMPLETE COORDINATES

Multi-Record with measure of "coordinates completeness of Multi-Records" equal "100%" are COMPLIANT with this Criterion.

Related Dimension in Context	Information Element	Resource Type
Coordinates Completeness of Multi-Records	Coordinates	Multi-Record

ALL THE MULTI-RECORD RECORDS MUST HAVE CONSISTENT COORDINATES

Data with measure of “coordinates consistency of Multi-Records" equal “100%” are COMPLIANT with this Criterion.

Related Dimension in Context	Information Element	Resource Type
Coordinates Consistency of Multi-Records	Coordinates	Multi-Record

ALL THE MULTI-RECORD RECORDS MUST HAVE COMPLETE SCIENTIFIC NAME

Data with measure of "scientific name completeness of Multi-Records" equal “100%" are COMPLIANT with this Criterion.

Related Dimension in Context	Information Element	Resource Type
Scientific Name Completeness of Multi-Records	Scientific Name	Multi-Record

ALL THE MULTI-RECORD RECORDS MUST HAVE CONFORM SCIENTIFIC NAME

Data with measure of “scientific name conformity of Multi-Records" equal “100%" are COMPLIANT with this Criterion.

Related Dimension in Context	Information Element	Resource Type
Scientific Name Conformity of Multi-Records	Scientific Name	Multi-Record

ALL THE MULTI-RECORD RECORDS MUST HAVE COMPLETE COLLECTED DATE

Data with measure of “collected date completeness of Multi-Records" equal “100%" are COMPLIANT with this Criterion.

Related Dimension in Context	Information Element	Resource Type
Collected Data Completeness of Multi-Records	Scientific Name	Multi-Record

ALL THE MULTI-RECORD RECORDS MUST HAVE CONSISTENT COLLECTED DATE

Data with measure of “collected date consistency of Multi-Records" equal “100%" are COMPLIANT with this Criterion.

Related Dimension in Context	Information Element	Resource Type
Collected Data Consistency of Multi-Records	Collected Date	Multi-Record

ALL THE MULTI-RECORD RECORDS MUST HAVE COMPLETE OCCURRENCE

Data with measure of “occurrence completeness of Multi-Records" equal “100%" are COMPLIANT with this Criterion.

Related Dimension in Context	Information Element	Resource Type
Occurrence Completeness of Multi-Records	Occurrence	Multi-Record

ALL THE MULTI-RECORD RECORDS MUST HAVE ACCURATE OCCURRENCE

Data with measure of "occurrence accuracy of Multi-Records" equal "100%" are COMPLIANT with this Criterion.

Related Dimension in Context	Information Element	Resource Type
Occurrence Accuracy of Multi-Records	Occurrence	Multi-Record

1.5. DQ Enhancement Policy

List of Enhancements that are particularly important for making data fitter for use in a particular Use Case context. The DQ Enhancement Policy has as goal to improve the measures of DQ Measurement Policy and consequently make data more compliant with the DQ Validation Policy.

RECOMMENDATION OF SCIENTIFIC NAME BASED ON NOMENCLATURE AUTHORITIES

DQ Enhancement	Information Element	Resource Type
Recommendation	Scientific Name	Single Record

RECOMMENDATION OF COLLECTED DATE BASED ON ISO 8601 STANDARD

DQ Enhancement	Information Element	Resource Type
Recommendation	Collected Date	Single Record

RECOMMENDATION OF COORDINATES BASED ON THE ASSOCIATED COUNTRY, STATE/PROVINCE, COUNTY AND LOCALITY

DQ Enhancement	Information Element	Resource Type
Recommendation	Coordinates	Single Record

ACCEPT ALL RECOMMENDATIONS FOR RECORDS IN THE MULTI-RECORD

DQ Enhancement	Information Element	Resource Type
Correction	Occurrence	Multi-Record

DISREGARD ALL RECORDS THAT ARE NOT COMPLIANT WITH ALL THE DQ CRITERIA

DQ Enhancement	Information Element	Resource Type

Correction	Occurrence	Multi-Record

2. DQ SOLUTIONS

2.1. Measurement Methods and Implementations

Description of how and what use to perform the DQ measure of each Dimension in context of the DQ Measurement Policy of the DQ profile. This description may have different formality levels depending the audience: for non-developers, this description supposed to be human readable but taking care to remove all ambiguity that might exist in the Dimension in context description; for developers, this description supposed to follow some standard to enable the software interoperability.

The following examples concern to provide a understandable description of measurements methods for a non-developer audience.

Dimension in Context	Specification	Mechanism
Coordinates Completeness of Single Records	Check if values (disregarding extra spaces) for both dwc:decimalLatitude and dwc:decimalLongitude are different to zero and different to empty. Applicable to Darwin Core based single records.	Name: FP-AKKA Kurator<br><br>Description: This software produces a data quality report on a set of natural science collections data. The software examines the data records for internal consistency, checks them against external services (such as Geolocate), identifies potential problems, and where possible, proposes corrections that may be applied to the data.<br><br>Institution: Kurator Project<br><br>URL: http://wiki.datakurator.net/web/Kurator

Coordinates Consistency of Single Records	Check if dwc:decimalLatitude and dwc:decimalLongitude values are consistent with coordinates ranges (latitudes range from -90 to 90 and longitudes range from -180 to 180) and are consistent with the bounds of dwc:country or close enough (currently defined as 200 km) from the georeference for dwc:country, dwc:stateProvince, dwc:county and dwc:locality. Applicable to Darwin Core based single records.<br><br>Georeference sources:<br>GeoLocate | Land data from Natural Earth | Country boundary data from GeoCommunity.	Name: FP-AKKA Kurator<br><br>Description: This software produces a data quality report on a set of natural science collections data. The software examines the data records for internal consistency, checks them against external services (such as Geolocate), identifies potential problems, and where possible, proposes corrections that may be applied to the data.<br><br>Institution: Kurator Project<br><br>URL: http://wiki.datakurator.net/web/Kurator
Scientific Name Completeness of Single Records	Check if values (disregarding extra spaces) for both dwc:scientificName and dwc:scientificNameAuthorship are different to empty. Applicable to Darwin Core based single records.	Name: FP-AKKA Kurator<br><br>Description: This software produces a data quality report on a set of natural science collections data. The software examines the data records for internal consistency, checks them against external services (such as Geolocate), identifies potential problems, and where possible, proposes corrections that may be applied to the data.<br><br>Institution: Kurator Project<br><br>URL: http://wiki.datakurator.net/web/Kurator
Scientific Name Conformity of Single Records	Check against nomenclature authorities if there is an exact match with both dwc:scientificName and dwc:scientificNameAuthorship values. Applicable to Darwin Core based single records.<br><br>Nomenclatural authority sources:<br>Catalog of Life | Global Name Resolver | Global Name Index | GBIF CheckListBank Backbone | IPNI | Index Fungorum | WoRMS.	Name: FP-AKKA Kurator<br><br>Description: This software produces a data quality report on a set of natural science collections data. The software examines the data records for internal consistency, checks them against external services (such as Geolocate), identifies potential problems, and where possible, proposes corrections that may be applied to the data.<br><br>Institution: Kurator Project<br><br>URL: http://wiki.datakurator.net/web/Kurator

Collected Date Completeness of Single Records	Check if value (disregarding extra spaces) for dwc:eventDate is different to empty. Applicable to Darwin Core based single records.	Name: FP-AKKA Kurator<br><br>Description: This software produces a data quality report on a set of natural science collections data. The software examines the data records for internal consistency, checks them against external services (such as Geolocate), identifies potential problems, and where possible, proposes corrections that may be applied to the data.<br><br>Institution: Kurator Project<br><br>URL: http://wiki.datakurator.net/web/Kurator
Collected Date Consistency of Single Records	Check if dwc:eventDate value is correctly formatted according to ISO 8601 standard and is before dwc:modified and within life span of dwc:collectedBy. Applicable to Darwin Core based single records.<br><br>Life span of collectors sources:<br>Harvard List of Botanists | FilteredPush Entomologists List.	Name: FP-AKKA Kurator<br><br>Description: This software produces a data quality report on a set of natural science collections data. The software examines the data records for internal consistency, checks them against external services (such as Geolocate), identifies potential problems, and where possible, proposes corrections that may be applied to the data.<br><br>Institution: Kurator Project<br><br>URL: http://wiki.datakurator.net/web/Kurator
Occurrence Completeness of Single Records	Check if values (disregarding extra spaces) for both dwc:decimalLatitude and dwc:decimalLongitude are different to zero or empty and values for dwc:eventDate, dwc:scientificName and dwc:scientificNameAuthorship are different to empty. Applicable to Darwin Core based single records.	Name: FP-AKKA Kurator<br><br>Description: This software produces a data quality report on a set of natural science collections data. The software examines the data records for internal consistency, checks them against external services (such as Geolocate), identifies potential problems, and where possible, proposes corrections that may be applied to the data.<br><br>Institution: Kurator Project<br><br>URL: http://wiki.datakurator.net/web/Kurator

Occurrence Accuracy of Single Records	Check if dwc:decimalLatitude and dwc:decimalLongitude values are consistent with coordinates ranges (latitudes range from -90 to 90 and longitudes range from -180 to 180) and are consistent with the bounds of dwc:country or close enough (currently defined as 200 km) to the georeference for dwc:country, dwc:stateProvince, dwc:county and dwc:locality and if dwc:eventDate value is correctly formatted according to ISO 8601 standard and is before dwc:modified and within life span of dwc:collectedBy and if there is an exact match with both dwc:scientificName and dwc:scientificNameAuthorship values with nomenclature authorities. Applicable to Darwin Core based single records.<br><br>Georeference sources:<br>GeoLocate | Land data from Natural Earth | Country boundary data from GeoCommunity.<br><br>Life span of collectors sources:<br>Harvard List of Botanists | FilteredPush Entomologists List.<br><br>Nomenclatural authority sources:<br>Catalog of Life | Global Name Resolver | Global Name Index | GBIF CheckListBank Backbone | IPNI | Index Fungorum | WoRMS.	Name: FP-AKKA Kurator<br><br>Description: This software produces a data quality report on a set of natural science collections data. The software examines the data records for internal consistency, checks them against external services (such as Geolocate), identifies potential problems, and where possible, proposes corrections that may be applied to the data.<br><br>Institution: Kurator Project<br><br>URL: http://wiki.datakurator.net/web/Kurator
Coordinates Completeness of Multi-Records	Calculate the proportion of records in Multi-Record with values (disregarding extra spaces) different to zero and different to empty for both dwc:decimalLatitude and dwc:decimalLongitude. Applicable to Multi-Records of Darwin Core based records.	Name: Biodiversity Data Quality Toolkit<br><br>Description: This toolkit provides an Application Programming Interface (API) for ad hoc use by applications that are able to consume Representational State Transfer (REST) web services. This toolkit provides mechanisms to measure and validate DQ of both Multi-Records and single records based on the Darwin Core standard.<br><br>Institution: Biocomp<br><br>URL: http://toolkit.bdq.biocomp.org.br:3020

Coordinates Consistency of Multi-Records	Calculate the proportion of records dwc:decimalLatitude and dwc:decimalLongitude values consistent with coordinates ranges (latitudes range from -90 to 90 and longitudes range from -180 to 180) and are consistent with the bounds of dwc:country or close enough (currently defined as 200 km) from the georeference for dwc:country, dwc:stateProvince, dwc:county and dwc:locality. Applicable to Multi-Records of Darwin Core based records.<br><br>Georeference sources:<br>GeoLocate | Land data from Natural Earth | Country boundary data from GeoCommunity.	Name: Biodiversity Data Quality Toolkit<br><br>Description: This toolkit provides an Application Programming Interface (API) for ad hoc use by applications that are able to consume Representational State Transfer (REST) web services. This toolkit provides mechanisms to measure and validate DQ of both Multi-Records and single records based on the Darwin Core standard.<br><br>Institution: Biocomp<br><br>URL: http://toolkit.bdq.biocomp.org.br:3020
Scientific Name Completeness of Multi-Records	Calculate the proportion of records in Multi-Record with values (disregarding extra spaces) different to empty for both dwc:scientificName and dwc:scientificNameAuthorship. Applicable to Multi-Records of Darwin Core based records.	Name: Biodiversity Data Quality Toolkit<br><br>Description: This toolkit provides an Application Programming Interface (API) for ad hoc use by applications that are able to consume Representational State Transfer (REST) web services. This toolkit provides mechanisms to measure and validate DQ of both Multi-Records and single records based on the Darwin Core standard.<br><br>Institution: Biocomp<br><br>URL: http://toolkit.bdq.biocomp.org.br:3020
Scientific Name Conformity of Multi-Records	Calculate the proportion of records with an exact match of both dwc:scientificName and dwc:scientificNameAuthorship values with nomenclatural authorities. Applicable to Multi-Records of Darwin Core based records.<br><br>Nomenclatural authority sources:<br>Catalog of Life | Global Name Resolver | Global Name Index | GBIF CheckListBank Backbone | IPNI | Index Fungorum | WoRMS.	Name: Biodiversity Data Quality Toolkit<br><br>Description: This toolkit provides an Application Programming Interface (API) for ad hoc use by applications that are able to consume Representational State Transfer (REST) web services. This toolkit provides mechanisms to measure and validate DQ of both Multi-Records and single records based on the Darwin Core standard.<br><br>Institution: Biocomp<br><br>URL: http://toolkit.bdq.biocomp.org.br:3020

Collected Date Completeness of Multi-Records	Calculate the proportion of records in Multi-Record that have the dwc:eventDate value (disregarding extra spaces) different to empty. Applicable to Multi-Records of Darwin Core based records.	Name: Biodiversity Data Quality Toolkit<br><br>Description: This toolkit provides an Application Programming Interface (API) for ad hoc use by applications that are able to consume Representational State Transfer (REST) web services. This toolkit provides mechanisms to measure and validate DQ of both Multi-Records and single records based on the Darwin Core standard.<br><br>Institution: Biocomp<br><br>URL: http://toolkit.bdq.biocomp.org.br:3020
Collected Date Consistency of Multi-Records	Calculate the proportion of dwc:eventDate value records that are correctly formatted according to ISO 8601 standard and that are before dwc:modified and within life span of collector. Applicable to Multi-Records of Darwin Core based records.<br><br>Life span of collectors sources: Harvard List of Botanists | FilteredPush Entomologists List.	Name: Biodiversity Data Quality Toolkit<br><br>Description: This toolkit provides an Application Programming Interface (API) for ad hoc use by applications that are able to consume Representational State Transfer (REST) web services. This toolkit provides mechanisms to measure and validate DQ of both Multi-Records and single records based on the Darwin Core standard.<br><br>Institution: Biocomp<br><br>URL: http://toolkit.bdq.biocomp.org.br:3020
Occurrence Completeness of Multi-Records	Calculate the proportion of records with values (disregarding extra spaces) for both dwc:decimalLatitude and dwc:decimalLongitude that are different to zero or empty and values for dwc:eventDate, dwc:scientificName and dwc:scientificNameAuthorship that are different to empty. Applicable to Multi-Records of Darwin Core based records.	Name: Biodiversity Data Quality Toolkit<br><br>Description: This toolkit provides an Application Programming Interface (API) for ad hoc use by applications that are able to consume Representational State Transfer (REST) web services. This toolkit provides mechanisms to measure and validate DQ of both Multi-Records and single records based on the Darwin Core standard.<br><br>Institution: Biocomp<br><br>URL: http://toolkit.bdq.biocomp.org.br:3020

Occurrence Accuracy of Multi-Records	Calculate the proportion of records in Multi-Record with values for dwc:decimalLatitude and dwc:decimalLongitude that are consistent with coordinates ranges (latitudes range from -90 to 90 and longitudes range from -180 to 180) and are consistent with the bounds of dwc:country or close enough (currently defined as 200 km) from the georeference for dwc:country, dwc:stateProvince, dwc:county and dwc:locality and value for dwc:eventDate is correctly formatted according to ISO 8601 standard and is before dwc:modified and within life span of dwc:collectedBy and there is an exact match with both dwc:scientificName and dwc:scientificNameAuthorship values with nomenclature authorities. Applicable to Darwin Core based single records.<br><br>Georeference sources:<br>GeoLocate | Land data from Natural Earth | Country boundary data from GeoCommunity.<br><br>Life span of collectors sources:<br>Harvard List of Botanists | FilteredPush Entomologists List.<br><br>Nomenclatural authority sources:<br>Catalog of Life | Global Name Resolver | Global Name Index | GBIF CheckListBank Backbone | IPNI | Index Fungorum | WoRMS.	Name: Biodiversity Data Quality Toolkit<br><br>Description: This toolkit provides an Application Programming Interface (API) for ad hoc use by applications that are able to consume Representational State Transfer (REST) web services. This toolkit provides mechanisms to measure and validate DQ of both Multi-Records and single records based on the Darwin Core standard.<br><br>Institution: Biocomp<br><br>URL: http://toolkit.bdq.biocomp.org.br:3020

2.2. Validation Methods and Implementations

Description of how and what use to perform the DQ validation of each Criterion of the DQ Validation Policy of the DQ profile. This description may have different formality levels depending the audience: for non-developers, this description supposed be human readable but taking care to remove any ambiguity existent in the Criterion description; for developers, this description supposed to follow some standard to enable the software interoperability.

The following examples concern to provide a comprehensive description of validations methods for a non-developer audience.

Criterion in Context	Specification	Mechanism

Coordinates must be complete	Check if values (disregarding extra spaces) for both dwc:decimalLatitude and dwc:decimalLongitude are different to zero and different to empty. Applicable to Darwin Core based single records.	Name: FP-AKKA Kurator<br><br>Description: This software produces a data quality report on a set of natural science collections data. The software examines the data records for internal consistency, checks them against external services (such as Geolocate), identifies potential problems, and where possible, proposes corrections that may be applied to the data.<br><br>Institution: Kurator Project<br><br>URL: http://wiki.datakurator.net/web/Kurator
Coordinates must be consistent	Check if dwc:decimalLatitude and dwc:decimalLongitude values are consistent with coordinates ranges (latitudes range from -90 to 90 and longitudes range from -180 to 180) and are consistent with the bounds of dwc:country or close enough (currently defined as 200 km) from the georeference for dwc:country, dwc:stateProvince, dwc:county and dwc:locality. Applicable to Darwin Core based single records.<br><br>Georeference sources:<br>GeoLocate | Land data from Natural Earth | Country boundary data from GeoCommunity.	Name: FP-AKKA Kurator<br><br>Description: This software produces a data quality report on a set of natural science collections data. The software examines the data records for internal consistency, checks them against external services (such as Geolocate), identifies potential problems, and where possible, proposes corrections that may be applied to the data.<br><br>Institution: Kurator Project<br><br>URL: http://wiki.datakurator.net/web/Kurator
Scientific Name must be complete	Check if values (disregarding extra spaces) for both dwc:scientificName and dwc:scientificNameAuthorship are different to empty. Applicable to Darwin Core based single records.	Name: FP-AKKA Kurator<br><br>Description: This software produces a data quality report on a set of natural science collections data. The software examines the data records for internal consistency, checks them against external services (such as Geolocate), identifies potential problems, and where possible, proposes corrections that may be applied to the data.<br><br>Institution: Kurator Project<br><br>URL: http://wiki.datakurator.net/web/Kurator

Scientific Name must be conform with nomenclatural authorities	Check against nomenclature authorities if there is an exact match with both dwc:scientificName and dwc:scientificNameAuthorship values. Applicable to Darwin Core based single records.<br><br>Nomenclatural authority sources:<br>Catalog of Life | Global Name Resolver | Global Name Index | GBIF CheckListBank Backbone | IPNI | Index Fungorum | WoRMS.	Name: FP-AKKA Kurator<br><br>Description: This software produces a data quality report on a set of natural science collections data. The software examines the data records for internal consistency, checks them against external services (such as Geolocate), identifies potential problems, and where possible, proposes corrections that may be applied to the data.<br><br>Institution: Kurator Project<br><br>URL: http://wiki.datakurator.net/web/Kurator
Collected Date must be complete	Check if value (disregarding extra spaces) for dwc:eventDate is different to empty. Applicable to Darwin Core based single records.	Name: FP-AKKA Kurator<br><br>Description: This software produces a data quality report on a set of natural science collections data. The software examines the data records for internal consistency, checks them against external services (such as Geolocate), identifies potential problems, and where possible, proposes corrections that may be applied to the data.<br><br>Institution: Kurator Project<br><br>URL: http://wiki.datakurator.net/web/Kurator
Collected Date must be consistent	Check if dwc:eventDate value is correctly formatted according to ISO 8601 standard and is before dwc:modified and within life span of dwc:collectedBy. Applicable to Darwin Core based single records.<br><br>Life span of collectors sources:<br>Harvard List of Botanists | FilteredPush Entomologists List.	Name: FP-AKKA Kurator<br><br>Description: This software produces a data quality report on a set of natural science collections data. The software examines the data records for internal consistency, checks them against external services (such as Geolocate), identifies potential problems, and where possible, proposes corrections that may be applied to the data.<br><br>Institution: Kurator Project<br><br>URL: http://wiki.datakurator.net/web/Kurator

Occurrence must be complete	Check if values (disregarding extra spaces) for both dwc:decimalLatitude and dwc:decimalLongitude are different to zero or empty and values for dwc:eventDate, dwc:scientificName and dwc:scientificNameAuthorship are different to empty. Applicable to Darwin Core based single records.	Name: FP-AKKA Kurator<br><br>Description: This software produces a data quality report on a set of natural science collections data. The software examines the data records for internal consistency, checks them against external services (such as Geolocate), identifies potential problems, and where possible, proposes corrections that may be applied to the data.<br><br>Institution: Kurator Project<br><br>URL: http://wiki.datakurator.net/web/Kurator
Occurrence must be accurate	Check if dwc:decimalLatitude and dwc:decimalLongitude values are consistent with coordinates ranges (latitudes range from -90 to 90 and longitudes range from -180 to 180) and are consistent with the bounds of dwc:country or close enough (currently defined as 200 km) to the georeference for dwc:country, dwc:stateProvince, dwc:county and dwc:locality and if dwc:eventDate value is correctly formatted according to ISO 8601 standard and is before dwc:modified and within life span of dwc:collectedBy and if there is an exact match with both dwc:scientificName and dwc:scientificNameAuthorship values with nomenclature authorities. Applicable to Darwin Core based single records.<br><br>Georeference sources:<br>GeoLocate | Land data from Natural Earth | Country boundary data from GeoCommunity.<br><br>Life span of collectors sources:<br>Harvard List of Botanists | FilteredPush Entomologists List.<br><br>Nomenclatural authority sources:<br>Catalog of Life | Global Name Resolver | Global Name Index | GBIF CheckListBank Backbone | IPNI | Index Fungorum | WoRMS.	Name: FP-AKKA Kurator<br><br>Description: This software produces a data quality report on a set of natural science collections data. The software examines the data records for internal consistency, checks them against external services (such as Geolocate), identifies potential problems, and where possible, proposes corrections that may be applied to the data.<br><br>Institution: Kurator Project<br><br>URL: http://wiki.datakurator.net/web/Kurator

All the Multi-Record records must have complete Coordinates	Check if the proportion of records in Multi-Record with values (disregarding extra spaces) different to zero and different to empty for both dwc:decimalLatitude and dwc:decimalLongitude is equal 100%. Applicable to Multi-Records of Darwin Core based records.	Name: Biodiversity Data Quality Toolkit<br><br>Description: This toolkit provides an Application Programming Interface (API) for ad hoc use by applications that are able to consume Representational State Transfer (REST) web services. This toolkit provides mechanisms to measure and validate DQ of both Multi-Records and single records based on the Darwin Core standard.<br><br>Institution: Biocomp<br><br>URL: http://toolkit.bdq.biocomp.org.br:3020
All the Multi-Record records must have consistent Coordinates	Check if the proportion of records dwc:decimalLatitude and dwc:decimalLongitude values consistent with coordinates ranges (latitudes range from -90 to 90 and longitudes range from -180 to 180) and are consistent with the bounds of dwc:country or close enough (currently defined as 200 km) from the georeference for dwc:country, dwc:stateProvince, dwc:county and dwc:locality is equal 100%. Applicable to Multi-Records of Darwin Core based records.<br><br>Georeference sources:<br>GeoLocate | Land data from Natural Earth | Country boundary data from GeoCommunity.	Name: Biodiversity Data Quality Toolkit<br><br>Description: This toolkit provides an Application Programming Interface (API) for ad hoc use by applications that are able to consume Representational State Transfer (REST) web services. This toolkit provides mechanisms to measure and validate DQ of both Multi-Records and single records based on the Darwin Core standard.<br><br>Institution: Biocomp<br><br>URL: http://toolkit.bdq.biocomp.org.br:3020
All the Multi-Record records must have complete Scientific Name	Check if the proportion of records in Multi-Record with values (disregarding extra spaces) different to empty for both dwc:scientificName and dwc:scientificNameAuthorship is equal 100%. Applicable to Multi-Records of Darwin Core based records.	Name: Biodiversity Data Quality Toolkit<br><br>Description: This toolkit provides an Application Programming Interface (API) for ad hoc use by applications that are able to consume Representational State Transfer (REST) web services. This toolkit provides mechanisms to measure and validate DQ of both Multi-Records and single records based on the Darwin Core standard.<br><br>Institution: Biocomp<br><br>URL: http://toolkit.bdq.biocomp.org.br:3020

All the Multi-Record records must have conform Scientific Name	Check if the proportion of records with an exact match of both dwc:scientificName and dwc:scientificNameAuthorship values with nomenclatural authorities is equal 100%. Applicable to Multi-Records of Darwin Core based records.<br><br>Nomenclatural authority sources:<br>Catalog of Life | Global Name Resolver | Global Name Index | GBIF CheckListBank Backbone | IPNI | Index Fungorum | WoRMS.	Name: Biodiversity Data Quality Toolkit<br><br>Description: This toolkit provides an Application Programming Interface (API) for ad hoc use by applications that are able to consume Representational State Transfer (REST) web services. This toolkit provides mechanisms to measure and validate DQ of both Multi-Records and single records based on the Darwin Core standard.<br><br>Institution: Biocomp<br><br>URL: http://toolkit.bdq.biocomp.org.br:3020
All the Multi-Record records must have complete Collected Date	Check if the proportion of records in Multi-Record that have the dwc:eventDate value (disregarding extra spaces) different to empty is equal 100%. Applicable to Multi-Records of Darwin Core based records.	Name: Biodiversity Data Quality Toolkit<br><br>Description: This toolkit provides an Application Programming Interface (API) for ad hoc use by applications that are able to consume Representational State Transfer (REST) web services. This toolkit provides mechanisms to measure and validate DQ of both Multi-Records and single records based on the Darwin Core standard.<br><br>Institution: Biocomp<br><br>URL: http://toolkit.bdq.biocomp.org.br:3020
All the Multi-Record records must have consistent Collected Date	Check if the proportion of dwc:eventDate value records that are correctly formatted according to ISO 8601 standard and that are before dwc:modified and within life span of collector is equal 100%. Applicable to Multi-Records of Darwin Core based records.<br><br>Life span of collectors sources:<br>Harvard List of Botanists | FilteredPush Entomologists List.	Name: Biodiversity Data Quality Toolkit<br><br>Description: This toolkit provides an Application Programming Interface (API) for ad hoc use by applications that are able to consume Representational State Transfer (REST) web services. This toolkit provides mechanisms to measure and validate DQ of both Multi-Records and single records based on the Darwin Core standard.<br><br>Institution: Biocomp<br><br>URL: http://toolkit.bdq.biocomp.org.br:3020

All the Multi-Record records must have complete Occurrence	Check if the proportion of records with values (disregarding extra spaces) for both dwc:decimalLatitude and dwc:decimalLongitude that are different to zero or empty and values for dwc:eventDate, dwc:scientificName and dwc:scientificNameAuthorship that are different to empty is equal 100%. Applicable to Multi-Records of Darwin Core based records.	Name: Biodiversity Data Quality Toolkit<br><br>Description: This toolkit provides an Application Programming Interface (API) for ad hoc use by applications that are able to consume Representational State Transfer (REST) web services. This toolkit provides mechanisms to measure and validate DQ of both Multi-Records and single records based on the Darwin Core standard.<br><br>Institution: Biocomp<br><br>URL: http://toolkit.bdq.biocomp.org.br:3020
All the Multi-Record records must have accurate Occurrence	Check if the proportion of records in Multi-Record with values for dwc:decimalLatitude and dwc:decimalLongitude that are consistent with coordinates ranges (latitudes range from -90 to 90 and longitudes range from -180 to 180) and are consistent with the bounds of dwc:country or close enough (currently defined as 200 km) from the georeference for dwc:country, dwc:stateProvince, dwc:county and dwc:locality and value for dwc:eventDate is correctly formatted according to ISO 8601 standard and is before dwc:modified and within life span of dwc:collectedBy and there is an exact match with both dwc:scientificName and dwc:scientificNameAuthorship values with nomenclature authorities is equal 100%. Applicable to Darwin Core based single records.<br><br>Georeference sources:<br>GeoLocate | Land data from Natural Earth | Country boundary data from GeoCommunity.<br><br>Life span of collectors sources:<br>Harvard List of Botanists | FilteredPush Entomologists List.<br><br>Nomenclatural authority sources:<br>Catalog of Life | Global Name Resolver | Global Name Index | GBIF CheckListBank Backbone | IPNI | Index Fungorum | WoRMS.	Name: Biodiversity Data Quality Toolkit<br><br>Description: This toolkit provides an Application Programming Interface (API) for ad hoc use by applications that are able to consume Representational State Transfer (REST) web services. This toolkit provides mechanisms to measure and validate DQ of both Multi-Records and single records based on the Darwin Core standard.<br><br>Institution: Biocomp<br><br>URL: http://toolkit.bdq.biocomp.org.br:3020

2.3. Enhancement Methods and Implementations

Description of how and what to use to perform the DQ Amendment of each Enhancement of the DQ Enhancement Policy of the DQ profile. This description may have different formality levels depending the audience: for non-developers, this description supposed be human readable but taking care to remove any ambiguity existent in the Enhancement description; for developers, this description supposed to follow some standard to enable the software interoperability.

The following examples concern to provide a comprehensive description of enhancement methods for a non-developer audience.

Enhancement in Context	Specification	Mechanism
Recommendation of Scientific Name based on nomenclature authorities	Recommend the most similar and valid Scientific Name and Scientific Name Authorship according to nomenclature authorities based on string similarity algorithms and in nomenclature rules and conventions. Details can be found at: http://sourceforge.net/p/filteredpush/svn/HEAD/tree/trunk/FP-Tools/FP-CurationServices/src/main/java/edu/harvard/mcz/nametools/. Applicable to Darwin Core based single records.<br><br>Nomencaltural authority sources: Catalog of Life | Global Name Resolver | Global Name Index | GBIF CheckListBank Backbone | IPNI | Index Fungorum | WoRMS.	Name: FP-AKKA Kurator<br><br>Description: This software produces a data quality report on a set of natural science collections data. The software examines the data records for internal consistency, checks them against external services (such as Geolocate), identifies potential problems, and where possible, proposes corrections that may be applied to the data.<br><br>Institution: Kurator Project<br><br>URL: http://wiki.datakurator.net/web/Kurator
Recommendation of Collected Date based on ISO 8601 standard	Recommend ISO 8601 standardized value for dwc:eventDate based on its own value, changing the order of year, month and day if they can be parsed or try to use dwc:year, dwc:month, dwc:day and dwc:startDayOfYear values to infer the corresponding dwc:evendDate value. Applicable to Darwin Core based single records.	Name: Biodiversity Data Quality Toolkit<br><br>Description: This toolkit provides an Application Programming Interface (API) for ad hoc use by applications that are able to consume Representational State Transfer (REST) web services. This toolkit provides mechanisms to measure and validate DQ of both Multi-Records and single records based on the Darwin Core standard.<br><br>Institution: Biocomp<br><br>URL: http://toolkit.bdq.biocomp.org.br:3020

Recommendation of Coordinates based on the associated country, state/province, county and locality	Change signs (positive and negative) of dwc:decimalLatitude and dwc:decimalLongitude and recommend the dwc:decimalLatitude and dwc:decimalLongitude that is consistent with dwc:country or consistent with the georeference of dwc:country, dwc:stateProvince, dwc:county and dwc:locality. Applicable to Darwin Core based single records.<br><br>Georeference sources: GeoLocate | Land data from Natural Earth | Country boundary data from GeoCommunity.	Name: FP-AKKA Kurator<br><br>Description: This software produces a data quality report on a set of natural science collections data. The software examines the data records for internal consistency, checks them against external services (such as Geolocate), identifies potential problems, and where possible, proposes corrections that may be applied to the data.<br><br>Institution: Kurator Project<br><br>URL: http://wiki.datakurator.net/web/Kurator

Accept all recommendations for records in the Multi-Record	For all records in Multi-Record, change values of dwc:scientificName and dwc:scientificNameAuthorship to the most similar and valid dwc:scientificName and dwc:scientificNameAuthorship according to nomenclature authorities based on string similarity algorithms and in nomenclature rules and conventions; change value of dwc:eventDate to an ISO 8601 standardized value based on its own value when it is possible to parse value into year, month and day or change dwc:eventDate to a date obtained from dwc:year, dwc:month, dwc:day and dwc:startDayOfYear values; and change values of dwc:decimalLatitude and dwc:decimalLongitude values with different signs (positive and negative) that is consistent with dwc:country or consistent with the georeference of dwc:country, dwc:stateProvince, dwc:county and dwc:locality. Applicable to Multi-Records of Darwin Core based records.<br><br>Georeference sources:<br>GeoLocate | Land data from Natural Earth | Country boundary data from GeoCommunity.<br><br>Life span of collectors sources:<br>Harvard List of Botanists | FilteredPush Entomologists List.<br><br>Nomenclatural authority sources:<br>Catalog of Life | Global Name Resolver | Global Name Index | GBIF CheckListBank Backbone | IPNI | Index Fungorum | WoRMS.	Name: Biodiversity Data Quality Toolkit<br><br>Description: This toolkit provides an Application Programming Interface (API) for ad hoc use by applications that are able to consume Representational State Transfer (REST) web services. This toolkit provides mechanisms to measure and validate DQ of both Multi-Records and single records based on the Darwin Core standard.<br><br>Institution: Biocomp<br><br>URL: http://toolkit.bdq.biocomp.org.br:3020

Disregard all records that are not compliant with all the DQ Criteria	Disregard records in Multi-Record with dwc:decimalLatitude and dwc:decimalLongitude values that are both equal to zero or one of them is equal to empty or they are not consistent with coordinates ranges (latitudes range from -90 to 90 and longitudes range from -180 to 180) and are not consistent with the bounds of dwc:country or not close enough (currently defined as 200 km) from the georeference for dwc:country, dwc:stateProvince, dwc:county and dwc:locality; or dwc:eventDate value is equal to empty or is not correctly formatted according to ISO 8601 standard or is not before dwc:modified or is not within life span of dwc:collectedBy; or there is not an exact match with both dwc:scientificName and dwc:scientificNameAuthorship values with nomenclature authorities or one of their values is empty. Applicable to Multi-Records of Darwin Core based records.<br><br>Georeference sources:<br>GeoLocate | Land data from Natural Earth | Country boundary data from GeoCommunity.<br><br>Life span of collectors sources:<br>Harvard List of Botanists | FilteredPush Entomologists List.<br><br>Nomenclatural authority sources:<br>Catalog of Life | Global Name Resolver | Global Name Index | GBIF CheckListBank Backbone | IPNI | Index Fungorum | WoRMS.	Name: Biodiversity Data Quality Toolkit<br><br>Description: This toolkit provides an Application Programming Interface (API) for ad hoc use by applications that are able to consume Representational State Transfer (REST) web services. This toolkit provides mechanisms to measure and validate DQ of both Multi-Records and single records based on the Darwin Core standard.<br><br>Institution: Biocomp<br><br>URL: http://toolkit.bdq.biocomp.org.br:3020

3. DQ Status

The DQ status is a list of assertions (DQ Measures, DQ Validations and DQ Amendments), created based on a particular DQ profile, that describes the current status of quality of a particular Data Resource. The DQ status supports data users and data owner to perform the fitness for use assessment and management according to the defined DQ profile.

We present two example of DQ reports organized into the following parts:

1. Data Resource: presents the Data Resources, where the first example presents a Single Record and the second example presents a Multi-Record.
2. DQ Measures: presents the DQ Measures assertions obtained for each Data Resource based on the DQ Dimensions defined in the DQ Measurement Policy and their respective DQ Solutions.
3. DQ Validations: presents the DQ Validations assertions obtained for each Data Resource based on the DQ Criteria defined in the DQ Validation Policy and their respective DQ Solutions.
4. DQ Amendment: presents the DQ Amendment assertions obtained for each Data Resource based on the DQ Enhancements defined in the DQ Enhancement Policy and their respective DQ Solutions.

3.1. DQ Status

3.1.1. DATA RESOURCE (Single Record)

Fields	Value
URL	http://case.bdq.biocomp.org.br:3010/api/v1.0/OrginalData/555f7b8ed53d8661fd3f53ed
dwc:eventDate	0000-00-00
dwc:municipality	Tijuana Municipality
dwc:oaiid	SCAN.occurrence.925670
dwc:identifiedBy	R.M. Bohart
dwc:georeferenceSources	Google Earth
dwc:geodeticDatum	WGS84
dwc:family	Vespidae
dwc:catalogNumber	ASUHIC0032157
dwc:recordedBy	C. Saheitlin
dwc:stateProvince	Baja California
dwc:year	1968
dwc:dateIdentified	1972
dwc:startDayOfYear	176
dwc:scientificName	Vespula pensylvanica
dwc:georeferenceVerificationStatus	requires verification
dwc:scientificNameAuthorship	Rohwer, 1857
dwc:ownerInstitutionCode	ASU
dwc:taxonID	http://api.gbif.org/v1/species/1311698
dwc:collectionCode	ASUHIC

dwc:modified	2013-12-05 20:02:40
dwc:country	Mexico
dwc:occurrenceRemarks	no collection date record
dwc:decimalLatitude	32.507822
dwc:basisOfRecord	PreservedSpecimen
dwc:institutionCode	ASU
dwc:decimalLongitude	-116.975289
dwc:month	6
dwc:locality	Los Angeles
dwc:day	24
dwc:georeferencedBy	David Fleming
id	555f7b8ed53d8661fd3f53ed

3.1.2. DQ MEASURES (Single Record)

Dimension in Context	Assertion	Specification	Mechanism
Coordinates Completeness of Single Records	COMPLETE	Check if values (disregarding extra spaces) for both dwc:decimalLatitude and dwc:decimalLongitude are different to zero and different to empty. Applicable to Darwin Core based single records.	Name: FP-AKKA Kurator
Coordinates Consistency of Single Records	NOT CONSISTENT	Check if dwc:decimalLatitude and dwc:decimalLongitude values are consistent with coordinates ranges (latitudes range from -90 to 90 and longitudes range from -180 to 180) and are consistent with the bounds of dwc:country or close enough (currently defined as 200 km) from the georeference for dwc:country, dwc:stateProvince, dwc:county and dwc:locality. Applicable to Darwin Core based single records.<br><br>Georeference sources:<br>GeoLocate | Land data from Natural Earth | Country boundary data from GeoCommunity.	Name: FP-AKKA Kurator
Scientific Name Completeness of Single Records	COMPLETE	Check if values (disregarding extra spaces) for both dwc:scientificName and dwc:scientificNameAuthorship are different to empty. Applicable to Darwin Core based single records.	Name: FP-AKKA Kurator

Scientific Name Conformity of Single Records	NOT CONFORM	Check against nomenclature authorities if there is an exact match with both dwc:scientificName and dwc:scientificNameAuthorship values. Applicable to Darwin Core based single records.<br><br>Nomenclatural authority sources:<br>Catalog of Life | Global Name Resolver | Global Name Index | GBIF CheckListBank Backbone | IPNI | Index Fungorum | WoRMS.	Name: FP-AKKA Kurator
Collected Date Completeness of Single Records	COMPLETE	Check if value (disregarding extra spaces) for dwc:eventDate is different to empty. Applicable to Darwin Core based single records.	Name: FP-AKKA Kurator
Collected Date Consistency of Single Records	NOT CONSISTENT	Check if dwc:eventDate value is correctly formatted according to ISO 8601 standard and is before dwc:modified and within life span of dwc:collectedBy. Applicable to Darwin Core based single records.<br><br>Life span of collectors sources:<br>Harvard List of Botanists | FilteredPush Entomologists List.	Name: FP-AKKA Kurator
Occurrence Completeness of Single Records	COMPLETE	Check if values (disregarding extra spaces) for both dwc:decimalLatitude and dwc:decimalLongitude are different to zero or empty and values for dwc:eventDate, dwc:scientificName and dwc:scientificNameAuthorship are different to empty. Applicable to Darwin Core based single records.	Name: FP-AKKA Kurator
Occurrence Accuracy of Single Records	NOT ACCURATE	Check if dwc:decimalLatitude and dwc:decimalLongitude values are consistent with coordinates ranges (latitudes range from -90 to 90 and longitudes range from -180 to 180) and are consistent with the bounds of dwc:country or close enough (currently defined as 200 km) to the georeference for dwc:country, dwc:stateProvince, dwc:county and dwc:locality and if dwc:eventDate value is correctly formatted according to ISO 8601 standard and is before dwc:modified and within life span of dwc:collectedBy and if there is an exact match with both dwc:scientificName and dwc:scientificNameAuthorship values with nomenclature authorities. Applicable to Darwin Core based single records.<br><br>Georeference sources:<br>GeoLocate | Land data from Natural Earth | Country boundary data from GeoCommunity.<br><br>Life span of collectors sources:<br>Harvard List of Botanists | FilteredPush Entomologists List.<br><br>Nomenclatural authority sources:<br>Catalog of Life | Global Name Resolver | Global Name Index | GBIF CheckListBank Backbone | IPNI | Index Fungorum | WoRMS.	Name: FP-AKKA Kurator

3.1.3. DQ VALIDATIONS
(Single Record)

Criterion in Context	Assertion	Specification	Mechanism
Coordinates must be complete	COMPLIANT	Check if values (disregarding extra spaces) for both dwc:decimalLatitude and dwc:decimalLongitude are different to zero and different to empty. Applicable to Darwin Core based single records.	Name: FP-AKKA Kurator
Coordinates must be consistent	NOT COMPLIANT	Check if dwc:decimalLatitude and dwc:decimalLongitude values are consistent with coordinates ranges (latitudes range from -90 to 90 and longitudes range from -180 to 180) and are consistent with the bounds of dwc:country or close enough (currently defined as 200 km) from the georeference for dwc:country, dwc:stateProvince, dwc:county and dwc:locality. Applicable to Darwin Core based single records.<br><br>Georeference sources:<br>GeoLocate | Land data from Natural Earth | Country boundary data from GeoCommunity.	Name: FP-AKKA Kurator
Scientific Name must be complete	COMPLIANT	Check if values (disregarding extra spaces) for both dwc:scientificName and dwc:scientificNameAuthorship are different to empty. Applicable to Darwin Core based single records.	Name: FP-AKKA Kurator
Scientific Name must be conform with nomenclatural authorities	NOT COMPLIANT	Check against nomenclature authorities if there is an exact match with both dwc:scientificName and dwc:scientificNameAuthorship values. Applicable to Darwin Core based single records.<br><br>Nomenclatural authority sources:<br>Catalog of Life | Global Name Resolver | Global Name Index | GBIF CheckListBank Backbone | IPNI | Index Fungorum | WoRMS.	Name: FP-AKKA Kurator
Collected Date must be complete	COMPLIANT	Check if value (disregarding extra spaces) for dwc:eventDate is different to empty. Applicable to Darwin Core based single records.	Name: FP-AKKA Kurator
Collected Date must be consistent	NOT COMPLIANT	Check if dwc:eventDate value is correctly formatted according to ISO 8601 standard and is before dwc:modified and within life span of dwc:collectedBy. Applicable to Darwin Core based single records.<br><br>Life span of collectors sources:<br>Harvard List of Botanists | FilteredPush Entomologists List.	Name: FP-AKKA Kurator

Occurrence must be complete	COMPLIANT	Check if values (disregarding extra spaces) for both dwc:decimalLatitude and dwc:decimalLongitude are different to zero or empty and values for dwc:eventDate, dwc:scientificName and dwc:scientificNameAuthorship are different to empty. Applicable to Darwin Core based single records.	Name: FP-AKKA Kurator
Occurrence must be accurate	NOT COMPLIANT	Check if dwc:decimalLatitude and dwc:decimalLongitude values are consistent with coordinates ranges (latitudes range from -90 to 90 and longitudes range from -180 to 180) and are consistent with the bounds of dwc:country or close enough (currently defined as 200 km) to the georeference for dwc:country, dwc:stateProvince, dwc:county and dwc:locality and if dwc:eventDate value is correctly formatted according to ISO 8601 standard and is before dwc:modified and within life span of dwc:collectedBy and if there is an exact match with both dwc:scientificName and dwc:scientificNameAuthorship values with nomenclature authorities. Applicable to Darwin Core based single records.<br><br>Georeference sources:<br>GeoLocate | Land data from Natural Earth | Country boundary data from GeoCommunity.<br><br>Life span of collectors sources:<br>Harvard List of Botanists | FilteredPush Entomologists List.<br><br>Nomenclatural authority sources:<br>Catalog of Life | Global Name Resolver | Global Name Index | GBIF CheckListBank Backbone | IPNI | Index Fungorum | WoRMS.	Name: FP-AKKA Kurator

3.1.4. DQ AMENDMENT
(Single Record)

Enhancement in Context	Assertion	Specification	Mechanism

Recommendation of Scientific Name based on nomenclature authorities	scientificName: "Vespula pensylvanica", scientificNameAuthorship: "(de Saussure, 1857)"	Recommend the most similar and valid Scientific Name and Scientific Name Authorship according to nomenclature authorities based on string similarity algorithms and in nomenclature rules and conventions. Details can be found at: http://sourceforge.net/p/filteredpush/svn/HEAD/tree/trunk/FP-Tools/FP-CurationServices/src/main/java/edu/harvard/mcz/nametools/. Applicable to Darwin Core based single records.<br><br>Nomencaltural authority sources:<br>Catalog of Life | Global Name Resolver | Global Name Index | GBIF CheckListBank Backbone | IPNI | Index Fungorum | WoRMS.	Name: FP-AKKA Kurator
Recommendation of Collected Date based on ISO 8601 standard	eventDate: "1968-06-24"	Recommend ISO 8601 standardized value for dwc:eventDate based on its own value, changing the order of year, month and day if they can be parsed or try to use dwc:year, dwc:month, dwc:day and dwc:startDayOfYear values to infer the corresponding dwc:evendDate value. Applicable to Darwin Core based single records.	Name: Biodiversity Data Quality Toolkit
Recommendation of Coordinates based on the associated country, state/province, county and locality	NO ASSERTION	Change signs (positive and negative) of dwc:decimalLatitude and dwc:decimalLongitude and recommend the dwc:decimalLatitude and dwc:decimalLongitude that is consistent with dwc:country or consistent with the georeference of dwc:country, dwc:stateProvince, dwc:county and dwc:locality. Applicable to Darwin Core based single records.<br><br>Georeference sources:<br>GeoLocate | Land data from Natural Earth | Country boundary data from GeoCommunity.	Name: FP-AKKA Kurator

3.2. DQ Status

3.2.1. DATA RESOURCE (Multi-Record)

Fields	Value
URL	http://case.bdq.biocomp.org.br:3010/api/v1.0/OrginalData
Number of Records	52411

3.2.2. DQ MEASURES (Multi-Record)

Dimension in Context	Assertion	Specification	Mechanism
Coordinates Completeness of Multi-Records	0.9999236815	Calculate the proportion of records in Multi-Record with values (disregarding extra spaces) different to zero and different to empty for both dwc:decimalLatitude and dwc:decimalLongitude. Applicable to Multi-Records of Darwin Core based records.	Name: Biodiversity Data Quality Toolkit
Coordinates Consistency of Multi-Records	0.8898153094	Calculate the proportion of records dwc:decimalLatitude and dwc:decimalLongitude values consistent with coordinates ranges (latitudes range from -90 to 90 and longitudes range from -180 to 180) and are consistent with the bounds of dwc:country or close enough (currently defined as 200 km) from the georeference for dwc:country, dwc:stateProvince, dwc:county and dwc:locality. Applicable to Multi-Records of Darwin Core based records.<br><br>Georeference sources:<br>GeoLocate | Land data from Natural Earth | Country boundary data from GeoCommunity.	Name: Biodiversity Data Quality Toolkit
Scientific Name Completeness of Multi-Records	0.9728115698	Calculate the proportion of records in Multi-Record with values (disregarding extra spaces) different to empty for both dwc:scientificName and dwc:scientificNameAuthorship. Applicable to Multi-Records of Darwin Core based records.	Name: Biodiversity Data Quality Toolkit
Scientific Name Conformity of Multi-Records	0.4928260703	Calculate the proportion of records with an exact match of both dwc:scientificName and dwc:scientificNameAuthorship values with nomenclatural authorities. Applicable to Multi-Records of Darwin Core based records.<br><br>Nomenclatural authority sources:<br>Catalog of Life | Global Name Resolver | Global Name Index | GBIF CheckListBank Backbone | IPNI | Index Fungorum | WoRMS.	Name: Biodiversity Data Quality Toolkit

Collected Date Completeness of Multi-Records	1	Calculate the proportion of records in Multi-Record that have the dwc:eventDate value (disregarding extra spaces) different to empty. Applicable to Multi-Records of Darwin Core based records.	Name: Biodiversity Data Quality Toolkit
Collected Date Consistency of Multi-Records	0.9670113714	Calculate the proportion of dwc:eventDate value records that are correctly formatted according to ISO 8601 standard and that are before dwc:modified and within life span of collector. Applicable to Multi-Records of Darwin Core based records.<br><br>Life span of collectors sources:<br>Harvard List of Botanists | FilteredPush Entomologists List.	Name: Biodiversity Data Quality Toolkit
Occurrence Completeness of Multi-Records	0.9727352514	Calculate the proportion of records with values (disregarding extra spaces) for both dwc:decimalLatitude and dwc:decimalLongitude that are different to zero or empty and values for dwc:eventDate, dwc:scientificName and dwc:scientificNameAuthorship that are different to empty. Applicable to Multi-Records of Darwin Core based records.	Name: Biodiversity Data Quality Toolkit
Occurrence Accuracy of Multi-Records	0.4286613752	Calculate the proportion of records in Multi-Record with values for dwc:decimalLatitude and dwc:decimalLongitude that are consistent with coordinates ranges (latitudes range from -90 to 90 and longitudes range from -180 to 180) and are consistent with the bounds of dwc:country or close enough (currently defined as 200 km) from the georeference for dwc:country, dwc:stateProvince, dwc:county and dwc:locality and value for dwc:eventDate is correctly formatted according to ISO 8601 standard and is before dwc:modified and within life span of dwc:collectedBy and there is an exact match with both dwc:scientificName and dwc:scientificNameAuthorship values with nomenclature authorities. Applicable to Darwin Core based single records.<br><br>Georeference sources:<br>GeoLocate | Land data from Natural Earth | Country boundary data from GeoCommunity.<br><br>Life span of collectors sources:<br>Harvard List of Botanists | FilteredPush Entomologists List.<br><br>Nomenclatural authority sources:<br>Catalog of Life | Global Name Resolver | Global Name Index | GBIF CheckListBank Backbone | IPNI | Index Fungorum | WoRMS.	Name: Biodiversity Data Quality Toolkit

3.2.3. DQ VALIDATIONS

(Multi-Record)

Criterion in Context	Assertion	Specification	Mechanism
All the Multi-Record records must have complete Coordinates	NOT COMPLIANT	Check if the proportion of records in Multi-Record with values (disregarding extra spaces) different to zero and different to empty for both dwc:decimalLatitude and dwc:decimalLongitude is equal 100%. Applicable to Multi-Records of Darwin Core based records.	Name: Biodiversity Data Quality Toolkit
All the Multi-Record records must have consistent Coordinates	NOT COMPLIANT	Check if the proportion of records dwc:decimalLatitude and dwc:decimalLongitude values consistent with coordinates ranges (latitudes range from -90 to 90 and longitudes range from -180 to 180) and are consistent with the bounds of dwc:country or close enough (currently defined as 200 km) from the georeference for dwc:country, dwc:stateProvince, dwc:county and dwc:locality is equal 100%. Applicable to Multi-Records of Darwin Core based records.<br><br>Georeference sources:<br>GeoLocate | Land data from Natural Earth | Country boundary data from GeoCommunity.	Name: Biodiversity Data Quality Toolkit
All the Multi-Record records must have complete Scientific Name	NOT COMPLIANT	Check if the proportion of records in Multi-Record with values (disregarding extra spaces) different to empty for both dwc:scientificName and dwc:scientificNameAuthorship is equal 100%. Applicable to Multi-Records of Darwin Core based records.	Name: Biodiversity Data Quality Toolkit
All the Multi-Record records must have conform Scientific Name	NOT COMPLIANT	Check if the proportion of records with an exact match of both dwc:scientificName and dwc:scientificNameAuthorship values with nomenclatural authorities is equal 100%. Applicable to Multi-Records of Darwin Core based records.<br><br>Nomenclatural authority sources:<br>Catalog of Life | Global Name Resolver | Global Name Index | GBIF CheckListBank Backbone | IPNI | Index Fungorum | WoRMS.	Name: Biodiversity Data Quality Toolkit
All the Multi-Record records must have complete Collected Date	COMPLIANT	Check if the proportion of records in Multi-Record that have the dwc:eventDate value (disregarding extra spaces) different to empty is equal 100%. Applicable to Multi-Records of Darwin Core based records.	Name: Biodiversity Data Quality Toolkit
All the Multi-Record records must have consistent Collected Date	NOT COMPLIANT	Check if the proportion of dwc:eventDate value records that are correctly formatted according to ISO 8601 standard and that are before dwc:modified and within life span of collector is equal 100%. Applicable to Multi-Records of Darwin Core based records.<br><br>Life span of collectors sources:<br>Harvard List of Botanists | FilteredPush Entomologists List.	Name: Biodiversity Data Quality Toolkit

All the Multi-Record records must have complete Occurrence	NOT COMPLIANT	Check if the proportion of records with values (disregarding extra spaces) for both dwc:decimalLatitude and dwc:decimalLongitude that are different to zero or empty and values for dwc:eventDate, dwc:scientificName and dwc:scientificNameAuthorship that are different to empty is equal 100%. Applicable to Multi-Records of Darwin Core based records.	Name: Biodiversity Data Quality Toolkit
All the Multi-Record records must have accurate Occurrence	NOT COMPLIANT	Check if the proportion of records in Multi-Record with values for dwc:decimalLatitude and dwc:decimalLongitude that are consistent with coordinates ranges (latitudes range from -90 to 90 and longitudes range from -180 to 180) and are consistent with the bounds of dwc:country or close enough (currently defined as 200 km) from the georeference for dwc:country, dwc:stateProvince, dwc:county and dwc:locality and value for dwc:eventDate is correctly formatted according to ISO 8601 standard and is before dwc:modified and within life span of dwc:collectedBy and there is an exact match with both dwc:scientificName and dwc:scientificNameAuthorship values with nomenclature authorities is equal 100%. Applicable to Darwin Core based single records.<br><br>Georeference sources:<br>GeoLocate | Land data from Natural Earth | Country boundary data from GeoCommunity.<br><br>Life span of collectors sources:<br>Harvard List of Botanists | FilteredPush Entomologists List.<br><br>Nomenclatural authority sources:<br>Catalog of Life | Global Name Resolver | Global Name Index | GBIF CheckListBank Backbone | IPNI | Index Fungorum | WoRMS.	Name: Biodiversity Data Quality Toolkit

3.2.4. DQ AMENDMENT
(Multi-Record)

Enhancement in Context	Assertion	Specification	Mechanism
Accept all recommendations for amendment of records in the Multi-Record (Data Quality Control)	URL: http://case.bdq.biocomp.org.br:3010/api/v1.0/DQReportControls<br><br>Number of Records: 52411	For all records in Multi-Record, change values of dwc:scientificName and dwc:scientificNameAuthorship to the most similar and valid dwc:scientificName and dwc:scientificNameAuthorship according to nomenclature authorities based on string similarity algorithms and in nomenclature rules and conventions; change value of dwc:eventDate to an ISO 8601 standardized value based on its own value when it is possible to parse value into year, month and day or change dwc:eventDate to a date obtained from dwc:year, dwc:month, dwc:day and dwc:startDayOfYear values; and change values of dwc:decimalLatitude and dwc:decimalLongitude values with different signs (positive and negative) that is consistent with dwc:country or consistent with the georeference of dwc:country, dwc:stateProvince, dwc:county and dwc:locality. Applicable to Multi-Records of Darwin Core based records.<br><br>Georeference sources:<br>GeoLocate | Land data from Natural Earth | Country boundary data from GeoCommunity.<br><br>Life span of collectors sources:<br>Harvard List of Botanists | FilteredPush Entomologists List.<br><br>Nomenclatural authority sources:<br>Catalog of Life | Global Name Resolver | Global Name Index | GBIF CheckListBank Backbone | IPNI | Index Fungorum | WoRMS.	Name: Biodiversity Data Quality Toolkit

Disregard all records that are not compliant with all the DQ Criteria (Data Quality Assurance)	URL: http://case.bdq.biocomp.org.br:3010/api/v1.0/DQReportAssurances<br><br>Number of Records: 32386	Disregard records in Multi-Record with dwc:decimalLatitude and dwc:decimalLongitude values that are both equal to zero or one of them is equal to empty or they are not consistent with coordinates ranges (latitudes range from -90 to 90 and longitudes range from -180 to 180) and are not consistent with the bounds of dwc:country or not close enough (currently defined as 200 km) from the georeference for dwc:country, dwc:stateProvince, dwc:county and dwc:locality; or dwc:eventDate value is equal to empty or is not correctly formatted according to ISO 8601 standard or is not before dwc:modified or is not within life span of dwc:collectedBy; or there is not an exact match with both dwc:scientificName and dwc:scientificNameAuthorship values with nomenclature authorities or one of their values is empty. Applicable to Multi-Records of Darwin Core based records.<br><br>Georeference sources:<br>GeoLocate | Land data from Natural Earth | Country boundary data from GeoCommunity.<br><br>Life span of collectors sources:<br>Harvard List of Botanists | FilteredPush Entomologists List.<br><br>Nomenclatural authority sources:<br>Catalog of Life | Global Name Resolver | Global Name Index | GBIF CheckListBank Backbone | IPNI | Index Fungorum | WoRMS.	Name: Biodiversity Data Quality Toolkit
