# Supplementary material for: A conceptual framework for quality assessment and management of biodiversity data
Source: PLoS One. 2017 Jun 28;12(6):e0178731. doi: 10.1371/journal.pone.0178731 (PMC5489162; doi:10.1371/journal.pone.0178731)
Supplement: S2 Appendix — The purpose of this document is to serve as quick reference for definitions of terms frequently used in this work. (PDF) [file pone.0178731.s002.pdf]

Glossary of Terms

DQ assessment: action of judging if DQ status makes data fit for use.

DQ management: action of improving DQ status for making data fitter for use.

DQ control: one of the approaches of DQ management, used for improving DQ when this is feasible.

DQ assurance: one of the approaches of DQ management, used for improving DQ in order to guarantee a satisfactory level of DQ, usually filtering data sets.

DQ Needs (class): class of concepts related to the definition of DQ meaning.

DQ Solutions (class): class of concepts related to DQ methods and tools used to meet DQ needs.

DQ Report (class): class of concepts related to the description of DQ status of a data resource.

Use Case: the context of use of data - describes an activity or process that uses data for a specific purpose.

Information Element (IE): is an abstraction that represents a relevant content in the Use Case context (e.g. coordinates).

DQ Dimension: measurable quality aspects (e.g. precision).

DQ Criterion: statement that describes acceptable levels of DQ (e.g value is in controlled vocabulary).

DQ Enhancement: statement that describes activities required to improve DQ.

Specification: a formal or informal description of a method for performing DQ measurements, validations or enhancements.

Mechanism: software, hardware, a technique, a tool, a person or any other artifact that implements one or more Specifications.

Data Resource: an instance of data.

Resource Type: type of Data Resource, which can be "single record" or "multi record".

DQ Assertion: a result of a measurement (of a DQ Dimension), a validation (according to a DQ Criterion) or an amendment (according to a DQ Enhancement), obtained by a Mechanism, operating on a specific Data Resource (single record or dataset), and using a specific method defined by a Specification.

DQ profile: defines the components necessary for enabling DQ assessment and management in the context of a Use Case.

DQ Measurement Policy: the component of DQ profile that describes a set of relevant DQ Dimensions for performing DQ measurements.

DQ Validation Policy: the component of DQ profile that describes a set of relevant DQ Criteria for performing DQ validations.

DQ Enhancement Policy: the component of DQ profile that describes a set of relevant DQ Enhancements for performing DQ enhancements.

DQ status: defines the status of quality of a Data Resource according to a DQ profile.

DQ Measures: part of DQ status that presents a set of measures of the DQ Dimensions defined in the DQ Measurement Policy of the related DQ profile.

DQ Validations: part of DQ status that presents a set of validations based on the DQ Criteria defined in the DQ Validation Policy of the related DQ profile.

DQ Amendments: part of DQ status that presents a set of amendments based on the DQ Enhancements defined in the DQ Enhancement Policy of the related DQ profile.

Fitness For Use Backbone (FFUB): proposal of a computational platform for registering and reusing components based on the conceptual framework, such as DQ profile (Use Cases, IEs, DQ Dimensions, DQ Criteria, DQ Enhancements), DQ status reports (DQ Assertions), DQ Solutions (Mechanism, Specification).
